# Exploring combinations of dimensionality reduction, transfer learning, and regularization methods for predicting binary phenotypes with transcriptomic data

**DOI:** 10.1186/s12859-024-05795-6

**Published:** 2024-04-26

**Authors:** S. R. Oshternian, S. Loipfinger, A. Bhattacharya, R. S. N. Fehrmann

**Affiliations:** Department of Medical Oncology, University Medical Center Groningen, University of Groningen, P.O. Box 30.001, 9700 RB Groningen, The Netherlands

**Keywords:** Dimensionality reduction, Transfer learning, Predictive modeling, Transcriptomic data, Independent component analysis, Autoencoder

## Abstract

**Background:**

Numerous transcriptomic-based models have been developed to predict or understand the fundamental mechanisms driving biological phenotypes. However, few models have successfully transitioned into clinical practice due to challenges associated with generalizability and interpretability. To address these issues, researchers have turned to dimensionality reduction methods and have begun implementing transfer learning approaches.

**Methods:**

In this study, we aimed to determine the optimal combination of dimensionality reduction and regularization methods for predictive modeling. We applied seven dimensionality reduction methods to various datasets, including two supervised methods (linear optimal low-rank projection and low-rank canonical correlation analysis), two unsupervised methods [principal component analysis and consensus independent component analysis (c-ICA)], and three methods [autoencoder (AE), adversarial variational autoencoder, and c-ICA] within a transfer learning framework, trained on > 140,000 transcriptomic profiles. To assess the performance of the different combinations, we used a cross-validation setup encapsulated within a permutation testing framework, analyzing 30 different transcriptomic datasets with binary phenotypes. Furthermore, we included datasets with small sample sizes and phenotypes of varying degrees of predictability, and we employed independent datasets for validation.

**Results:**

Our findings revealed that regularized models without dimensionality reduction achieved the highest predictive performance, challenging the necessity of dimensionality reduction when the primary goal is to achieve optimal predictive performance. However, models using AE and c-ICA with transfer learning for dimensionality reduction showed comparable performance, with enhanced interpretability and robustness of predictors, compared to models using non-dimensionality-reduced data.

**Conclusion:**

These findings offer valuable insights into the optimal combination of strategies for enhancing the predictive performance, interpretability, and generalizability of transcriptomic-based models.

**Supplementary Information:**

The online version contains supplementary material available at 10.1186/s12859-024-05795-6.

## Introduction

There is an ongoing effort to develop predictive models that utilize transcriptomic profiles. Clinicians can use such models to select, for example, the optimal treatment for each patient, i.e., precision medicine. Unfortunately, only a few transcriptomic-based predictive models have reached clinical practice, e.g., for oncology, these include Oncotype DX and MammaPrint [1]. One reason for this limited adaptation is that the sample size is often too small compared to the numerous potential input predictors—in this case, gene expression levels—used to train the predictive model. As a result, overfitting can occur when a model learns the details and noise in the training dataset to such an extent that it negatively impacts the model's performance on new data.

Dimensionality reduction or regularization techniques can be employed to mitigate overfitting [2]. Well-known unsupervised dimensionality reduction methods include principal component analysis (PCA), consensus independent component analysis (c-ICA), and autoencoders (AE) [3–5]. Both PCA and c-ICA linearly transform a training dataset with many predictors into a new set—comprising fewer predictors—that still retains most of the information in the original dataset. In PCA and c-ICA, the new predictors are the activity scores (loading factors or mixing matrix weights, respectively) of each component in each sample. An AE is a type of deep neural network that consists of an encoder and a decoder network. The encoder network learns to reduce the data's dimensionality, transforming numerous predictors into a limited set of new predictors (i.e., latent representation). The decoder network learns to reconstruct the input data from these latent representations with minimal loss of information. Supervised dimensionality reduction methods, such as linear optimal low-rank projection (LOL) and low-rank canonical correlation analysis (CCA), effectively reduce data to a lower-dimensional representation, maintaining class-related information [6, 7]. These methods differ from unsupervised approaches by utilizing both predictor variables and class labels to inform the reduction process.

Unsupervised dimensionality reduction methods could benefit from transfer learning, a technique that enables them to draw on insights gained from more extensive and diverse datasets [8]. In our approach, we leveraged a comprehensive set of transcriptomic profiles—beyond those used for predictive model training—to refine the c-ICA and AE dimensionality reduction methods. The refined c-ICA linear transformation, or the AE's encoder network, is subsequently applied to a specific dataset to generate a new set of predictors. These predictors are then used as input to train the predictive model. Employing transfer learning in this manner has the potential to increase the robustness of these new predictors, thereby mitigating overfitting and enhancing the model's predictive performance.

Lasso, Ridge, and Elastic Net are popular regularization techniques used to mitigate overfitting [9–11]. During the training phase, these techniques aim to minimize the impact of predictors that are highly correlated with each other, thereby reducing model complexity. Generally, these regularization techniques add a penalty to the model as its complexity increases during training. Combining dimensionality reduction methods with these regularization techniques might even further improve the model's predictive performance.

**Fig. 1:**
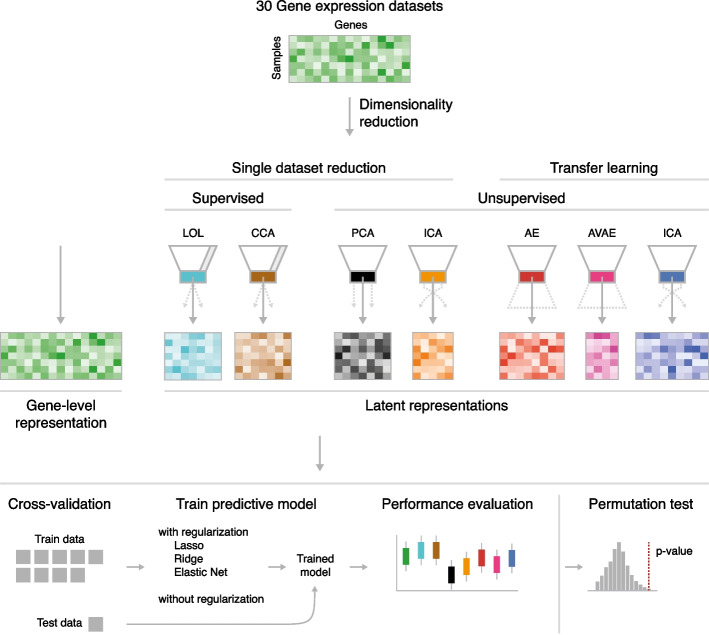
Study overview. Preprocessed gene expression datasets served as input for two supervised dimensionality reduction methods: linear optimal low-rank projection (LOL) and low-rank canonical correlation analysis (CCA), alongside two unsupervised methods: principal component analysis (PCA) and consensus independent component analysis (c-ICA). Furthermore, latent representations were generated using a transfer learning approach with an autoencoder (AE), adversarial variational autoencoder (AVAE), and c-ICA, all trained on the GPL570 dataset. These gene-level and latent representations were then individually utilized in the predictive modeling pipeline, employing a cross-validation strategy with and without three different regularization techniques to evaluate predictive performance. The statistical significance of model performance was determined using a permutation test

In this study, our aim was to determine which dimensionality reduction method across supervised approaches (LOL, CCA), unsupervised approaches (PCA, c-ICA), and transfer learning approaches (AE, AVAE, and c-ICA) can enhance predictive performance of models. We investigated their impact on predictive performance with and without the application of regularization techniques. For this, we trained predictive models on 30 different transcriptomic datasets and their dimensionality-reduced counterparts. We then evaluated the models' performance and the robustness of predictor selection using a cross-validation setup encapsulated in a permutation testing framework (Fig. 1).

## Methods

### Data acquisition of an extensive compendium of transcriptomic profiles (GPL570 dataset)

Publicly available raw gene expression data generated using the Affymetrix HG-U133 Plus 2.0 microarray platform was obtained from the Gene Expression Omnibus (GEO; accession number GPL570) [12]. Preprocessing and aggregation of raw gene expression data were conducted using the robust multi-array average algorithm available in Analysis Power Tools (release 2.11.3). The mapping of probesets to genes and quality control procedures are described in Additional file 1: Supplementary Note. This comprehensive collection of transcriptomic profiles is referred to as the GPL570 dataset.

For predictive analysis, we selected transcriptomic datasets with available biological phenotypes from GEO. All selected datasets were preprocessed in the same manner as the GPL570 dataset.

### Training of the autoencoder

Autoencoder (AE) methods are a type of unsupervised deep neural network utilized for various tasks, including feature learning, dimensionality reduction, data denoising, and classification [5]. The encoder network learns to reduce the dimensionality of the input data by mapping it to a latent space, thereby generating a new representation with a limited number of variables, referred to as the latent representation. The decoder network learns to reconstruct the samples in the input data from their latent representations with minimal loss of information.

In our study, the encoder maps the GPL570 dataset, consisting of 139,786 samples and 19,863 genes, to a latent representation with 1024 latent variables. The gene expression levels serve as the input variables for the encoder. The decoder attempts to reconstruct the samples' gene expression levels from their latent representations. A schematic representation of the AE can be found in Additional file 1: Fig. S1, and a detailed description of hyperparameters and layers is provided in Additional file 1: Table S1.

After randomly shuffling the samples in the GPL570 dataset, we divided the dataset into training (70%, n = 97,850), validation (15%, n = 20,968), and test sets (15%, n = 20,968). The training of AEs is focused on minimizing the difference between the input and reconstructed data. We employed the Mean Squared Error (MSE) loss function to train the encoder and the decoder parameters. AE training was conducted using the Ranger optimizer [13]. The MSE was used to train and evaluate the model's performance from a sample perspective. In addition, we calculated a metric to gauge the reconstruction performance from the gene perspective. First, we calculated the Pearson correlation between the genes in the input data, resulting in a triangular correlation matrix with dimension *p* genes by *p* genes. Second, we calculated the same triangular correlation matrix using the reconstructed data. We then computed the absolute difference between the correlations obtained with the input and the reconstructed data for each gene pair. After sorting the absolute correlation differences in ascending order, we obtained the 95th percentile, referred to as the R-difference^95th^, as a reconstruction performance metric from the gene perspective. The closer the R-difference^95th^ is to zero, the better the reconstructed data captures the gene-by-gene correlation structure present in the input data.

### Training of the adversarial variational autoencoder

An adversarial variational autoencoder (AVAE) is a type of deep neural network that enhances the capabilities of a standard AE by incorporating adversarial training and imposing a constraint on the latent distribution [14]. This approach can result in more biologically meaningful representations in the latent space and may allow, for example, the generation of new transcriptomic profiles with similar properties by adding noise to the latent representation [15]. A schematic representation of the AVAE and a detailed description of its hyperparameters and layers is provided in Additional file 1: Fig. S2 and Table S2.

To train and evaluate the AVAE, we used the same GPL570 training, validation, and test sets as with the AE. During the training process, the AVAE aims to encode the samples' gene expression levels into a latent representation and to reconstruct them from this latent representation. In our AVAE, we imposed constraints that force the latent variables to conform to a Gaussian prior distribution. We employed the Ranger optimizer, using parameters identical to those in the AE training, to minimize both the MSE for reconstruction performance and the Kullback–Leibler divergence (KL) to regulate the distribution of the latent variables [16].

The calculation of loss for the encoder and decoder is as follows: The encoder loss is the sum of the MSE loss of the reconstructed data and the KL loss of the generated latent representation. The decoder loss is the sum of the discriminator loss for the reconstructed data, the discriminator loss for the generated data, and the MSE loss of the reconstructed data.

### Consensus independent component analysis on the GPL570 dataset

Consensus independent component analysis (c-ICA) was performed to segregate bulk transcriptomic profiles into statistically independent transcriptional components (TCs), as previously described in more detail [17].

In brief, applying c-ICA to a transcriptomic dataset with *p* genes and *n* samples yields *i* transcriptional components, each of dimension 1 × *p*. Each TC captures the transcriptional pattern of an underlying process (e.g., biological process or cellular state). A TC comprises *p* scalars, representing both the direction and magnitude of the effect of the underlying process on a gene's expression level. In addition to the TCs, c-ICA also outputs a mixing matrix (MM) of dimension *i* × *n*, containing coefficients for each TC-sample pair. The inner product between an individual sample's vector of coefficients in the MM and the vector of scalars for each gene across all TCs results in reconstructed transcriptomic profiles that closely resemble the input profiles. Thus, the coefficients in the MM can be interpreted in a similar manner to the latent representations derived from the AE and AVEA.

Initially, a preprocessing technique called whitening is applied to the input dataset to accelerate the convergence rate of the ICA algorithm. This involves conducting PCA on the sample-by-sample covariance matrix. The number of principal components capturing at least 90% of the total variance in the GPL570 dataset serves as the input for the c-ICA. Due to the random initialization of the optimization algorithm, 25 ICA runs were conducted, and only TCs that could be identified consistently across multiple runs were selected using a consensus approach. For parameters and details on performing the c-ICA algorithm on the GPL570 dataset, please refer to Additional file 1: Supplementary Note. The c-ICA yield TCs, each representing a robust, statistically independent, and distinct transcriptional pattern of an underlying process, along with a mixing matrix that describes the latent representations of the transcriptomes.

### Dimensionality reduction methods applied to datasets

Various dimensionality reduction methods were applied to each transcriptomic dataset. For the purpose of reference comparison, all datasets were also included without any dimensionality reduction. These datasets, which contain a high number of genes and thus feature complexity, are hereafter referred to as gene-level datasets. The gene-level datasets served as input for several dimensionality reduction methods, which are described below.

#### Supervised dimensionality reduction

Supervised dimensionality reduction effectively reduces data to a lower-dimensional representation, maintaining class-related information. We applied two supervised methods, linear optimal low-rank projection (LOL) and low-rank canonical correlation analysis (CCA), to gene-level datasets with their corresponding phenotype classes [6, 7]. While LOL uses class-conditional means and class-centered covariance to optimize data representation for improved classification, CCA focuses on identifying correlated patterns between samples from specific phenotype classes. The resulting representations are referred to as LOL and CCA latent representations, respectively.

#### Principal component analysis

Principal component analysis (PCA) is a widely used method for reducing the dimensionality of transcriptomic data [3]. In each gene-level dataset, individual gene's expression levels were standardized to have a mean of 0 and a standard deviation of 1. The sample correlation matrix was then calculated. Subsequently, the eigenvectors and eigenvalues of this matrix were determined. The pseudo-inverse of the eigenvector matrix yields activity scores for each principal component (PC) in each sample. These activity scores, capturing 100% of the variance observed in the gene-level dataset, are referred to as the single PCA latent representation.

#### Consensus independent component analysis

c-ICA was trained independently on each of the gene-level datasets. For every dataset, the number of principal components that accounted for 100% of the dataset variance during the whitening step was used as the input for ICA. Only TCs that were identified multiple times across 50 runs were selected, following a consensus approach (see Additional file 1: Supplementary Note for details). The resulting mixing matrix, which comprises the activity scores for each TC across all samples, is referred to as the single ICA latent representation.

#### Autoencoder

We utilized each gene-level dataset as input for the AE network, which had been pre-trained on the GPL570 dataset. Our objective in applying transfer learning was focused on dimensionality reduction, rather than transferring specific phenotypic information. To achieve a compact representation, we passed the datasets through the encoder layers of the AE. This resulting representation, encompassed by 1024 latent variables, is referred to as the AE latent representation.

#### Adversarial variational autoencoder

Each gene-level dataset was individually used as input for the trained AVAE network. The datasets were processed through both the shared and the specific encoder networks, resulting in a representation with 256 latent variables (refer to Additional file 1: Fig. S2 for details). This latent representation contains vectors representing the mean and standard deviation. This mean vector consists of 128 latent variables for each sample and is referred to as the AVAE latent representation.

#### c-ICA transformation obtained with the GPL570 dataset

Each gene-level dataset was projected into its latent representation using the c-ICA model that had been trained on the GPL570 dataset. This involved calculating the pseudo-inverse of the independent component matrix and then multiplying this by the matrix of the gene-level dataset [18]. The resulting projected mixing matrix for each dataset included activity scores for 3286 independent components per sample, captured as latent variables, and is referred to as the GPL570 ICA latent representation.

In summary, we generated seven different representations for each gene-level dataset (see Fig. 1). First, we applied dimensionality reduction methods on each dataset individually using two supervised methods to obtain 'LOL' and 'CCA' latent representations, and two unsupervised methods to construct 'single PCA' and 'single ICA' latent representations. Secondly, we leveraged unsupervised transfer learning approaches, using three models trained on the GPL570 dataset: an AE, an AVAE, and a c-ICA model, yielding three additional latent representations: 'AE', 'AVAE', and 'GPL570 ICA'. The seven representations, alongside the original gene-level data, formed the basis for our subsequent predictive modeling.

### Predictive modeling

#### Predictive models without regularization

In our predictive modeling, logistic regression without regularization was utilized for phenotype prediction. The disproportionately large number of predictors compared to the sample size, rendered the direct application of a logistic regression model infeasible. To circumvent this issue, we employed the Median Absolute Deviation (MAD) to identify the most variable predictors, thereby capping the number of predictors to match the sample size. Predictors exhibiting the highest MAD were deemed to indicate the most significant variation among samples, thus more likely to discern differences in the data. These specifically chosen predictors were subsequently used to train the logistic regression model.

#### Predictive models using regularization techniques

We also incorporated logistic regression combined with three regularization techniques: Lasso, Ridge, and Elastic Net, for phenotype prediction [7–9, 19]. These techniques are designed to deal with correlated predictors and provide more stable models. Lasso produces sparse models by setting the coefficients of certain predictors to zero. Ridge assigns coefficients close to zero to reduce multicollinearity. Elastic Net combines the strengths of both Lasso and Ridge. These techniques help us identify informative input predictors with minimal inter-correlation for effective prediction. Detailed information with all parameters can be found in Additional file 1: Supplementary Note.

#### Evaluation metrics for performance

To assess the predictive performance of the models, the Matthew Correlation Coefficient (MCC) was selected as the primary evaluation metric to [20]. In binary classification context, the MCC functions as an equivalent to the discrete version of the Pearson correlation coefficient and is interpreted in a similar manner [21]. In addition, to draw more robust conclusions on the predictive performance, we also included the Adjusted Rand Index (ARI) and the 1-Brier score [22, 23]. Note that the MCC is considered more informative than both the ARI and the Brier Score in binary classification evaluations [24]. When regularization techniques were applied, the definitive metric score was established by identifying the highest performance value from among the three regularization techniques: Lasso, Ridge, and Elastic Net.

#### Cross-validation

To validate the performance of the predictive models, a *k*-fold cross-validation (CV) was used. Initially, the samples in a dataset were divided into *k* folds in a stratified manner based on the phenotypic labels. Subsequently, *k* − 1 of these folds were used for training, while the remaining *k*_th_ fold served as the test set. After completing the CV, the overall model performance was calculated using the aggregated predictive labels from all folds. CV was conducted in two different ways: first with *k* = 10, resulting in a 90% training and 10% testing split; and second, in a reverse scheme with *k* = 5, leading to a 20% training and 80% testing split, to examine the model's predictive behavior when trained on a smaller sample size.

#### Permutation test

A phenotype permutation test was conducted using 200 permutations to evaluate the significance of the predictive model's performance. Prior to carrying out the CV, the phenotypic labels were randomly shuffled. For each of these random reshufflings, a cross-validated performance metric was calculated using the predictive model, thereby generating a null distribution of the model's predictive performance. The statistical significance for testing the null hypothesis—that there is no association between the input predictors and the phenotypic label—was indicated by a p-value. This p-value is defined as the proportion of permutations yielding an performance metric equal or better than the metric obtained with non-permutated phenotypic labels, relative to the total number of permutations. The final reported performance metric is the mean value of metric across the 200 CV runs with the non-permutated phenotypic labels. We refer to this combination of CV and permutation testing as the CV-permutation test.

#### Regularization technique comparison

We conducted a paired samples Wilcoxon test to assess whether one regularization technique significantly outperforms the others when applied with specific dimensionality reduction methods. For each dimensionality reduction method, the performance difference between regularization techniques were tested across 30 datasets. The resulting p-values were subsequently adjusted using the Bonferroni correction for multiple testing.

#### Robustness of input predictors

To evaluate the robustness of predictor selection within the prediction models, we used a cross-validation approach focused specifically on Lasso regularization. Unlike Ridge and Elastic Net, Lasso has the ability to zero out predictors, that do not increase predictive performance. For each dataset, we conducted 20 CV runs, using stratified sampling to divide the data into ten folds for each run. During each CV run, we trained ten separate Lasso regression models on unique combinations of nine folds. We then identified predictors with non-zero coefficients in these Lasso models for further analysis. The robustness of these predictors was evaluated by examining all ten models from each of the 20 CV runs, culminating in a comprehensive assessment over 200 individual models. Due to the variable number of predictors across different representations, we used the proportion of occurrence for each predictor, calculated as the number of times a predictor had a non-zero coefficient divided by the total number of predictors that had a non-zero coefficient in at least one CV run. The robustness of the predictor selection was then quantified by calculating the area under the curve for the proportion of predictors relative to the number of runs (AUC of proportion). A higher AUC of proportion indicates greater robustness in predictor selection. Furthermore, robustness was also assessed utilized the pairs of independent datasets with identical phenotype classes. A Lasso model was trained separately on each dataset within a pair. We then calculated the robustness by determining the proportion of predictors that were selected by both Lasso models in the pair compared to all selected predictors.

## Results

### Extensive compendium of transcriptomic profiles

To implement the transfer learning approach in our dimensionality reduction methods, we collected 139,786 transcriptomic profiles, which encompass measurements for 19,863 unique genes (GPL570 dataset). From this comprehensive dataset, we selected 30 studies that featured binary phenotypes to conduct predictive modeling. Among these 30 studies are five pairs of independent datasets, each investigating the same phenotype. The sample size in these selected studies ranged from 46 to 437, and they spanned a diverse array of phenotypes (see Additional file 1: Table S3).

### AE, AVAE, and c-ICA can effectively reduce ~ 140 K transcriptomic profiles to latent representations

We aimed to evaluate the efficacy of AE, AVAE, and c-ICA as dimensionality reduction methods in transforming the GPL570 dataset's transcriptomic profiles into lower dimensional latent representations, while minimizing information loss.

For the AE method, we chose the network configuration at epoch 540, based on its performance metrics with the validation set (see Additional file 1: Fig. S3 for learning curves). When applied to the test set, the AE network displayed an MSE of 0.0965 and R-difference^95th^ of 0.0310. These low values indicate both accurate reconstruction of the transcriptomic profiles and retention of the gene-by-gene correlation structure. Validation test further confirmed that the AE network was not prone to overfitting (refer to Table 1). The trained AE network is available in Supplementary Data 1.

**Table 1:** Final autoencoder (AE) and adversarial variational autoencoder (AVAE) performance on the test and validation set

	Reconstructed			Generated		
	MSE	R-difference^95th^	KL	MSE	R-difference^95th^	KL
*AE*						
Test	0.0965	0.0310	–	–	–	–
Validation	0.0966	0.0305	–	–	–	–
*AVAE*						
Test	0.1968	0.0922	0.1071	–	0.1256	0.1028
Validation	0.1971	0.0927	0.1035	–	0.1266	0.1032

In the case of AVAE, we selected the network at epoch 7500. Compared to the AE network, the AVAE network displayed a slightly lower reconstruction performance, with an MSE of 0.1968 and an R-difference^95th^ of 0.0922. Also, for this network, no overfitting was observed (Table 1; Additional file 1: Fig. S3). This relatively lower performance is attributed to the network's design, which aims to normalize the latent variables to a normal distribution, as indicated by a KL value of 0.1071. The performance metric values for the generator network showed that the latent space could be successfully used to generate new profiles with a gene-by-gene correlation structure similar to that observed in the profiles of the validation and test sets (validation R-difference^95th^ = 0.1266). In this study, we only used the decoder network of the AVAE. The trained AVAE network is provided as Supplementary Data 2.

As for c-ICA, the method generated latent representations with 3286 latent variables, where a latent variable corresponds to the activity score of a TC. Its reconstruction performance, measured by an MSE of 0.3578 and an R-difference^95th^ of 0.2490, was lower compared to both AE and AVAE. This decrease in performance is due to c-ICA's stricter constraint of enforcing statistical independence among the latent variables. The c-ICA latent variables can be found in Supplementary Data 3.

In summary, our results indicate that AE, AVAE, and c-ICA are effective at reducing the dimensionality of transcriptomic profiles to latent representations, albeit with varying degrees of information loss.

### A broad range of predictability of phenotypes across 30 datasets

**Fig. 2:**
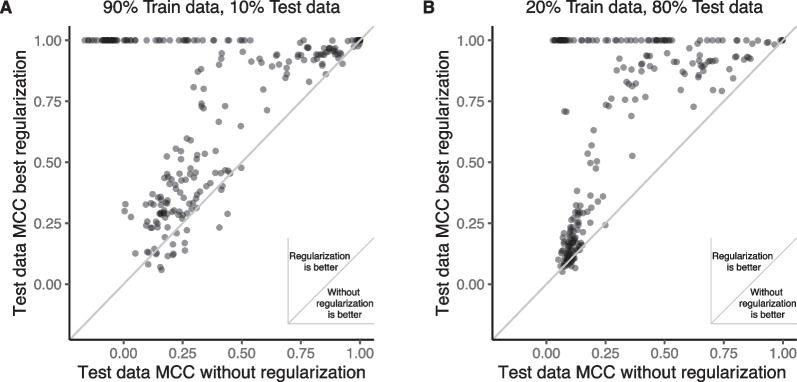
Comparative analysis of predictive model performance with and without regularization. The performance of predictive models applied to gene-level data and their latent representations across 30 datasets is shown. The performance is displayed as the test data Matthew correlation coefficient (MCC) obtained through the CV-permutation test for both the predictive model with the best regularization technique and the model without regularization. The CV was executed with two distinct settings: **A** using 90% of the data samples for training and **B** utilizing 20% for training

We explored the predictive capabilities of our predictive models across a variety of biological phenotypes. In comparisons of predictive models with and without regularization, we observed mostly superior performance from models employing regularization, as evidenced by their MCC, ARI, and Brier Score metrics (see Fig. 2A; Additional file 1: Fig. S4). This effect was especially marked in datasets with smaller sample sizes, where models without regularization were more prone to overfitting (Fig. 2B). Consequently, we are directing our focus towards the performance of prediction models that incorporate regularization. Our models' performance using the gene—level representations, as primarily assessed by the MCC, indicated a broad spectrum of phenotype predictability. For example, certain phenotypes like leukemia type (GSE131184 with an MCC of 0.97) and differentiation between normal and cancer tissues (GSE35570 with an MCC of 1.0 and GSE53757 with an MCC of 0.94) were highly predictable using gene-level representations. Conversely, other phenotypes such as renal transplant rejection (GSE36059 with an MCC of 0.54 and GSE48581 with an MCC of 0.45), and detection of Parkinson disease from blood samples (GSE99039 with an MCC of 0.29) exhibited lower predictability. Additional details of latent representations and predictive performances can be found in Supplementary Data 4 and Supplementary Data 5, respectively. The additional performance metrics we considered, ARI and Brier scores, demonstrated a strong correlation with MCC (Spearman's rho: ARI = 0.94, Brier score = 0.88), as shown in Additional file 1: Fig. S5. They also mirrored the MCC in terms of variability in predictive performance. This variation in predictive performance metrics allows us to further investigate how different modeling strategies affect performance across various levels of phenotype predictability.

### Supervised dimensionality reduction: Promising predictive performance on training data but less reliable on truly independent datasets

**Fig. 3:**
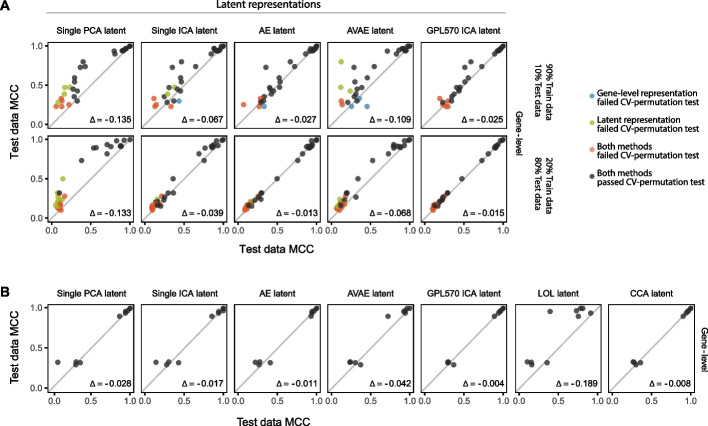
Comparative predictive performance of latent representations and gene-level representation. **A** The Matthews correlation coefficient (MCC) for the most effective regularization technique across all 30 datasets is presented. The top row shows cross-validation performance with 90% of the data used for training, while the bottom row shows performance with only 20% used for training. The mean MCC difference (Δ) between gene-level and latent representations is indicated, with a negative Δ value signifying better performance of the gene-level representation. **B** The predictive performance of models with regularization trained on latent representations is displayed across five pairs of independent datasets. Within each pair, one dataset was used for training predictive models, and the paired dataset served as the test set for performance assessment

To evaluate the capacity of latent representations from supervised dimensionality reduction methods to enhance predictive performance, we applied LOL and CCA to each dataset. These methods produced new predictors by incorporating the phenotype labels during the reduction process, which significantly improved the performance of subsequent regression models, as shown in Additional file 1: Figs. S6–S8 A, B. However, this apparent high performance may be due to information leakage during the dimensionality reduction phase. Since phenotype labels were used to guide the reduction across all samples, potential bias in the cross-validation results may occur. This 'bleeding' of information between the training and test folds could lead to an overestimation of the true predictive performance of the models. This concern was substantiated when we evaluated the models on a truly independent dataset with the same phenotype. While in general, a performance drop was observed in the independent dataset, CCA managed to yield similar predictive performance as gene-level data (mean MCC difference of − 0.009). In contrast, the LOL latent representation underperformed, with a mean MCC difference of − 0.189 compared to the gene-level representation, suggesting a greater tendency for overfitting as evidenced by its lower performance on a truly independent dataset (as detailed in Fig. 3B). This pattern was consistent for both the ARI and the Brier score (as detailed in Additional file 1: Figs. S6–S8C). In essence, although predictive models utilizing these supervised dimensionality reduction techniques show promise on the datasets they were trained on, their performance may not be as reliable when applied to novel, independent datasets.

### Dimensionality reduction methods PCA and c-ICA led to lower predictive performance

We examined the impact of using latent representations with fewer predictors generated through PCA or c-ICA on the performance of predictive models, compared to using gene-level representations. The mean MCC differences when using single PCA and single ICA latent representations compared to gene-level representations were − 0.135 and − 0.067, respectively (refer to Fig. 3A). This indicated diminished performance for both methods, with PCA showing a more substantial decrease in performance relative to gene-level representations across almost all datasets. This pattern is similar for both the ARI and the Brier score (refer to Additional file 1: Figs. S6–S8). To ascertain the statistical significance of these performances, we performed a CV-permutation test. The MCC scores were significant for most datasets across all three methods; however, PCA's MCC scores were not significant for 10 out of the 30 datasets (as shown in Fig. 3A). In summary, our results suggest that employing PCA and c-ICA for dimensionality reduction did not enhance the predictive models' performance when used in conjunction with regularization techniques, and in some instances led to reduced performance.

### Dimensionality reduction methods AE and c-ICA combined with transfer learning showed comparable predictive performance compared to models using gene-level data

We investigated the potential for enhanced predictive performance when employing latent representations derived from transfer learning approaches using AE, AVAE, and c-ICA, in comparison to using gene-level representations. Our analysis revealed that the mean MCC differences between the latent representations obtained through AE, AVAE, and GPL570 ICA and the gene-level representations were − 0.027, − 0.109, and − 0.025, respectively (as shown in Fig. 3A). While AE and GPL570 ICA yielded slightly diminished performance relative to gene-level representations, the data suggest that these transfer learning approaches effectively preserve the essential information even when the number of predictors is reduced. Conversely, the AVAE latent representation captured less relevant information than its gene-level, AE latent, and GPL570 ICA latent counterparts. Significance results through CV-permutation test further validated that the observed performances were not obtained by chance. Consistent with our CV performance findings, we noted parallel outcomes across the five pairs of independent datasets (see Fig. 3B). This consistency was also evident for both the ARI and Brier Score metrics (see Additional file 1: Figs. S6–S8). Details on all dataset representations and CV-permutation test results can be found in Supplementary Data 4 and Supplementary Data 5, respectively. A pairwise comparison between all representations is illustrated in Additional file 1: Fig. S9.

In summary, our results demonstrate that employing transfer learning approaches to project transcriptomic profiles into more compact, lower-dimensional representations succeeds in preserving the biological information relevant to specific phenotypes, all while reducing the number of predictors required in the models.

### Dimensionality reduction methods are more effective for datasets with small sample sizes

We explored the efficacy of predictive models on datasets with smaller sample sizes, utilizing only 20% of the available samples, which ranged from 9 to 87 in number. In general, a notable decrease in predictive performance was observed, and a higher proportion of datasets failed to show significant predictability according to the CV-permutation test. Nevertheless, predictive models with datasets showcasing a more marked phenotypic divergence, such as comparisons between cancerous and healthy tissues (GSE35570, GSE53757), demonstrated more resilient performance (see Additional file 1: Table S4). Importantly, the mean MCC differences between the gene-level and latent representations became more modest. This trend suggests that dimensionality reduction methods tend to perform comparably to gene-level representations in scenarios involving smaller sample sizes. Hence, our results imply that dimensionality reduction methods are more beneficial when dealing with datasets that have limited sample sizes compared to those with more extensive sample collections.

### The predictive performance is more dependent on phenotype than on regularization techniques

In order to investigate whether there exists a 'best-fit' regularization technique for each type of data representation, we assessed the MCC difference between various regularization techniques across all 30 datasets. Our analyses revealed that, for most data representations, no single regularization technique consistently outperformed the others within each dataset (refer to Additional file 1: Table S5 and Fig. S10). However, there were some specific cases: for the AE latent representation, Ridge was frequently the optimal choice; for PCA, both Lasso and Elastic Net tended to be more effective than Ridge; and for gene-level data, Elastic Net often surpassed Lasso. Importantly, we found that the effect of choosing a specific regularization technique was comparatively minor when set against the inherent predictability of the phenotype in each dataset.

### Dimensionality reduction leads to more stable selection of input predictors

**Fig. 4:**
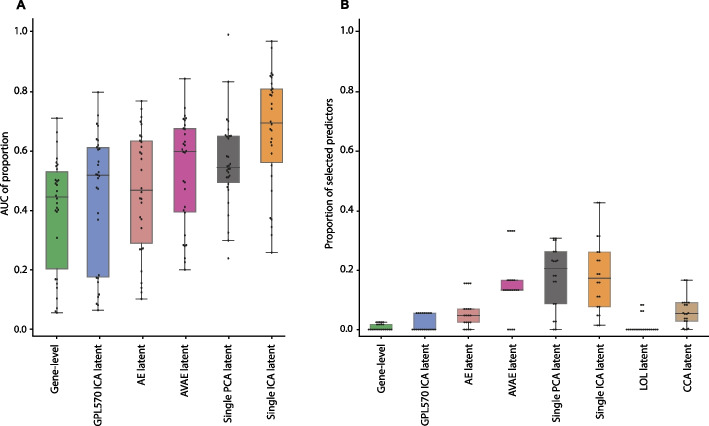
Robustness of predictor selection across various representations. **A** The area under the curve (AUC) of proportion of selected predictors is displayed for each representation across all 30 datasets. The hinges of the boxes denote the second and third quartiles, and the whiskers extend by half of that interquartile range. The center of each box represents the median value. **B** The proportion of selected predictors for five pairs of independent datasets is displayed for each representation

To assess whether dimensionality reduction methods lead to a more stable selection of input predictors, we calculated the AUC of proportion for each dataset across various reduction methods (refer to Fig. 4A; Additional file 1: Fig. S11 and Data S6). For example, in the GSE64951 dataset with the single ICA latent representation, the AUC of proportion was 0.97, suggesting that most input predictors were consistently selected across all runs. In contrast, the same dataset's gene-level representation had an AUC of proportion of only 0.15, despite having comparable MCC values (gene-level MCC = 0.43, single ICA latent MCC = 0.45). While variability existed across datasets, the single ICA latent representation generally exhibited the most robust selection of predictors (see Additional file 1: Table S6). Conversely, the gene-level representation often demonstrated less consistency in predictor selection.

Overall, as the number of runs increased, the proportion of selected predictors decreased. Nevertheless, certain key predictors were consistently chosen across the 200 models, highlighting their significance in the predictive models.

In analyses of predictor selection robustness for models trained on pairs of independent datasets, we noted a similar trend as described above (refer to Fig. 4B). Models based on the supervised methods CCA and LOL exhibited low robustness in predictor selection. When these methods were used to train predictive models on new datasets, multiple predictors, including the primary discriminative ones, were frequently selected. This indicates that CCA and LOL might preferentially capture dataset-specific information as the main discriminative predictors and distribute phenotype-specific information among various predictors.

## Discussion

In this study, we utilized 30 transcriptomic datasets to determine the optimal combination of dimensionality reduction methods, transfer learning approaches, and regularization techniques to achieve the most effective predictive models. We found that predictive models trained directly on transcriptomic data, without employing dimensionality reduction (i.e., using gene-level representation), yielded the highest predictive performance across multiple metrics. However, the models utilizing the AE and GPL570 ICA latent representations of the datasets exhibited almost similar levels of performance and exhibited improved interpretability and robustness in predictor selection compared to models using gene-level representations.

Dimensionality reduction methods can effectively mitigate overfitting of predictive models by reducing the complexity of input data, eliminating noise and irrelevant information, and focusing on the most informative aspects [25, 26]. Moreover, dimensionality reduction methods improve generalization by capturing the underlying structure or patterns in the data, enabling the model to better generalize to unseen instances and reducing the likelihood of overfitting to specific training examples [26].

While the abundance of predictors in gene-level representations presents a modeling challenge (i.e. overfitting), they inherently encapsulate the complete spectrum of phenotypic information necessary for accurate prediction. Our results indicate that predictive models trained with regularization techniques can already effectively extract the phenotypic information and mitigate overfitting, even when dealing with many potential predictors as in the gene-level representations. The combination of dimensionality reduction methods and regularization techniques did not yield further improvements in predictive performance. While both methods independently reduce the risk of overfitting, our results show that they do not necessarily enhance each other's ability to boost predictive performance.

The comparable performance observed between predictive models utilizing the gene-level representation and the latent representations obtained through GPL570 ICA or AE highlights the effectiveness of these transfer learning approaches combined with dimensionality reduction methods in capturing the phenotype-relevant information inherent in the gene-level data. This finding is consistent across a broad range of phenotype predictability, further emphasizing the robustness and utility of these methods. It is worth noting that GPL570 ICA exhibited a lower reconstruction performance compared to AE, which can be attributed to the less than 100% explained variance and the imposed statistical independence restriction on the TCs in GPL570 ICA. This suggests that excellent reconstruction performance is not necessarily a prerequisite for effectively capturing phenotype-relevant information in a latent representation.

While models using GPL570 ICA and AE representations have already demonstrated high predictive performance, there remains room for potential improvement. For instance, optimizing the AE network structure or increasing the explained variance to obtain more TCs from c-ICA could enhance performance, provided that computational resources permit. These optimizations could capture additional phenotype-relevant information within the latent representation. Such enhancements have the potential to further elevate the performance of models using these methods' representations, making their performance even more comparable to models using the gene-level representation.

Differences in predictive performance between dimensionality reduction methods may be due to the inherent characteristics and limitations of each method, which affect how the data is reduced in dimensionality. PCA reduces data complexity by creating new orthogonal axes that spread the data as much as possible, thereby capturing most of the observed variance with fewer new predictors. c-ICA reduces dimensionality with the constraint of statistical independence—stricter than orthogonality—which minimizes any shared information between new predictors. This minimized shared information may explain the higher predictive performance observed when c-ICA is used compared to PCA. Furthermore, c-ICA has been shown to reveal more subtle and biologically relevant patterns in the data compared to PCA, which is particularly useful in scenarios involving complex signals or mixtures (i.e., the bulk transcriptomic profiles generated from complex tissue biopsies used in this study) [27]. In the context of AEs, the AVAE regularizes the latent distribution to follow a Gaussian distribution, which has been shown to improve the generative ability and interpretability, but diminish the predictive performance compared to an AE without this regularization. Techniques like AEs and c-ICA can benefit from transfer learning, utilizing a diverse collection of samples to effectively uncover complex biological patterns, typically leading to more robust new predictors. Conversely, training AEs and c-ICA on a single dataset may only reflect the unique biological patterns within that dataset, yielding new predictors that are more specific for that dataset but potentially less generalizable to other datasets. It is also important to recognize that transfer learning approaches, while robust, are more demanding in terms of resources and might overlook unique biological details not represented in their extensive training sets. Transfer learning for the supervised dimensionality reduction is not feasible on the same scale as for unsupervised, as in the majority of samples the required labels used in the reduction process will not be available.

In addition to predictive performance, the interpretability of predictive models plays a crucial role in understanding the underlying phenotype-relevant biological processes. When dealing with phenotypes primarily driven by a limited number of genes, gene-level representation is appropriate as all the phenotype-relevant genes have a high chance of being selected as input predictors [28]. However, for phenotypes involving intricate gene interactions and diverse biological processes, the complexity can lead to variability in input predictor selection. In such cases, capturing this complexity with dimensionality reduction methods in a latent representation with a lower number of predictors offers the advantage of a more robust selection, resulting in higher generalizability and interpretability. For AE, various network interpretation methods can aid in identifying which genes have the most influence on each latent variable selected as an input predictor [29]. In c-ICA, each TC captures a statistically independent transcriptional pattern, often associated with a specific biological process, which can be identified using gene set enrichment analysis [30]. Thus, AE and c-ICA have the advantage of providing a more robust and interpretable latent representation, enabling a deeper understanding of the underlying biological mechanisms.

To our knowledge, our study is the most comprehensive comparative analysis with the aim to determine which optimal combination of dimensionality reduction method across supervised approaches, unsupervised approaches, transfer learning, and regularization techniques can enhance the predictive performance of models. Two other studies used transcriptomic data to investigate the impact of dimensionality reduction on the performance of predictive models [31, 32]. One study used a single breast cancer dataset to show that the classification accuracy of a support vector machine (SVM) for estrogen receptor status decreased to varying degrees for several dimensionality reduction methods, including PCA [31]. The other study used a highly imbalanced dataset (498 cancer samples and 52 non-cancer samples) to analyze the impact of different dimensionality reduction methods (PCA, kernel PCA, and autoencoder) on machine learning models (neural network and SVM) used for cancer prediction (non-cancer versus cancer). The F-measures reported in their study reveal only marginal differences in performance [32]. In contrast to these studies, we used a cross-validation setup encapsulated within a permutation testing framework, analyzed 30 different transcriptomic datasets, included datasets with small sample sizes and phenotypes of varying degrees of predictability, and we utilized truly independent datasets for validation.

In conclusion, the results from this comprehensive comparative study indicate that when prioritizing predictive performance, utilizing gene-level data in predictive modeling with regularization techniques yields the best results. Dimensionality reduction with PCA or c-ICA on the dataset itself yielded suboptimal predictive performance. However, when combined with transfer learning, dimensionality reduction methods like c-ICA and AE showed predictive performance comparable to that of gene-level data. Additionally, these methods offered advantages in terms of predictor selection's reproducibility and interpretability.

### Supplementary Information

**Additional file 1.** Supplementary Tables, Supplementary Figures, and Supplementary Note.

## Data Availability

Microarray expression data were collected from the public Gene Expression Omnibus repository under the accession number GPL570 (generated using the Affymetrix HG-U133 Plus 2.0 platform). All Supplementary Data and codes required to reproduce the results are available at the following link: https://zenodo.org/records/10404690.
