# Supplementary material for: Exploring combinations of dimensionality reduction, transfer learning, and regularization methods for predicting binary phenotypes with transcriptomic data
Source: BMC Bioinformatics. 2024 Apr 26;25:167. doi: 10.1186/s12859-024-05795-6 (PMC11046904; doi:10.1186/s12859-024-05795-6)
Supplement: Supplementary file 1 — Additional file 1. Supplementary Tables, Supplementary Figures, and Supplementary Note. [file 12859_2024_5795_MOESM1_ESM.docx]

**SUPPLEMENTARY TABLES**

**Supplementary Table 1.** *Structure and hyperparameters of the AE network.*

| **Network models** | **Hyper parameter** | | | | | | |
| --- | --- | --- | --- | --- | --- | --- | --- |
|  | **Hidden units’ size** | **Bias** | **Kernel Initialization** | **Optimizer** | **Learning rate** | **Warm up** | **Batch size** |
| **Encoder** | Dense 12288 | 0.1 | He Normal | Ranger | 0.005 | 0.1 | 32 |
|  | LN(layer normalization) |  |  |  |  |  |  |
|  | Dense 8192 | 0.1 | He Normal |  |  |  |  |
|  | LN |  |  |  |  |  |  |
|  | Dense 4096 | 0.1 | He Normal |  |  |  |  |
|  | LN |  |  |  |  |  |  |
|  | Dense 2048 | 0.1 | He Normal |  |  |  |  |
|  | LN |  |  |  |  |  |  |
|  | Dense 1024 | 0.1 | Glorot uniform |  |  |  |  |
| **Decoder** | Dense 2048 | 0.1 | He Normal | Ranger | 0.005 | 0.1 | 32 |
|  | LN |  |  |  |  |  |  |
|  | Dense 4096 | 0.1 | He Normal |  |  |  |  |
|  | LN |  |  |  |  |  |  |
|  | Dense 8192 | 0.1 | He Normal |  |  |  |  |
|  | LN |  |  |  |  |  |  |
|  | Dense 12288 | 0.1 | He Normal |  |  |  |  |
|  | LN |  |  |  |  |  |  |
|  | Dense 19863 | 0.1 | Glorot uniform |  |  |  |  |

**Supplementary Table 2.** *Structure and hyperparameters of AVAE network.*

| **Network models** | **Hyper parameter** | | | | | | |
| --- | --- | --- | --- | --- | --- | --- | --- |
|  | **Hidden units’ size** | **Bias** | **Kernel Initialization** | **Optimizer** | **Learning rate** | **Warm up** | **Batch size** |
| **Shared Encoder** | Dense 4098 | 0.1 | He Normal | Ranger | 5.00E-03 | 0.1 | 32 |
|  | LN(layer normalization) |  |  |  |  |  |  |
|  | Dense 2048 | 0.1 | He Normal |  |  |  |  |
|  | LN |  |  |  |  |  |  |
|  | Dense 512 | 0.1 | He Normal |  |  |  |  |
|  | LN |  |  |  |  |  |  |
|  | Dense 128 | 0.1 | Glorot uniform |  |  |  |  |
|  | LN |  |  |  |  |  |  |
| **Specific Encoder** | Dense 256 | 0.1 | Glorot uniform | Ranger | 5.00E-03 | 0.1 | 32 |
| **Decoder** | Dense 512 | 0.1 | He Normal | Ranger | 5.00E-03 | 0.1 | 32 |
|  | LN |  |  |  |  |  |  |
|  | Dense 2048 | 0.1 | He Normal |  |  |  |  |
|  | LN |  |  |  |  |  |  |
|  | Dense 2048 | 0.1 | He Normal |  |  |  |  |
|  | LN |  |  |  |  |  |  |
|  | Dense 19863 |  | Glorot uniform |  |  |  |  |
| **Discriminators** | Dense 64 | 0.1 | He Normal | Ranger | 5.00E-03 | 0.1 | 32 |
|  | LN |  |  |  |  |  |  |
|  | Dense 32 | 0.1 | He Normal |  |  |  |  |
|  | LN |  |  |  |  |  |  |
|  | Dense 16 | 0.1 | He Normal |  |  |  |  |
|  | LN |  |  |  |  |  |  |
|  | Dense 1 | 0.1 | Glorot uniform |  |  |  |  |

**Supplementary Table 3**. *Characteristics of datasets used to train predictive models.*

| **dataset** | **number of samples** | **predictive phenotype** | **phenotype distribution** |
| --- | --- | --- | --- |
| GSE117239 | 60 | lesion_tissue | 32 x non-lesion, 28 x psoriasis |
| GSE131184 | 68 | leukemia_type | 39 x AML, 29 x T-ALL |
| GSE13159 | 313 | leukemia_type | 202 x AML, 111 x T-ALL |
| GSE14067 | 54 | was_rejected | 27 x FALSE, 27 x TRUE |
| GSE20685 | 327 | event_metastasis | 244 x 0, 83 x 1 |
| GSE20910 | 49 | has_down_syndrome | 26 x FALSE, 23 x TRUE |
| GSE22459 | 65 | was_rejected | 25 x FALSE, 40 x TRUE |
| GSE28571 | 56 | survival | 26 x long, 30 x short |
| GSE30674 | 56 | is_control | 44 x FALSE, 12 x TRUE |
| GSE30999 | 67 | lesion_tissue | 34 x non-lesion, 33 x psoriasis |
| GSE31189 | 92 | disease_state | 52 x bladder_cancer, 40 x no_bladder_cancer |
| GSE33113 | 89 | recurrence_3years | 71 x no, 18 x yes |
| GSE33630 | 105 | tissue_type | 60 x cancer, 45 x normal_histology |
| GSE35570 | 116 | tissue_type | 65 x cancer, 51 x normal_histology |
| GSE36059 | 247 | was_rejected | 183 x FALSE, 64 x TRUE |
| GSE37199 | 94 | stage | 63 x advanced_castration_resistant, 31 x good_prognosis |
| GSE39582 | 288 | MSI_HIGH_VS_MSO | 57 x MSI, 231 x MSO |
| GSE48581 | 300 | was_rejected | 222 x FALSE, 78 x TRUE |
| GSE50006 | 90 | leukemia_type | 69 x CLL, 21 x healthy B-cells |
| GSE51024 | 96 | tissue_type | 55 x cancer, 41 x normal |
| GSE53757 | 144 | tissue_type | 72 x clear cell renal cell carcinoma,  72 x normal kidney |
| GSE56311 | 54 | condition | 29 x Diagnosis, 25 x Relapse 1 |
| GSE58812 | 107 | event_metastasis | 76 x 0, 31 x 1 |
| GSE60542 | 91 | is_multifocal | 53 x no, 38 x yes |
| GSE64951 | 94 | disease_state | 63 x gastric_cancer, 31 x normal |
| GSE66161 | 74 | tissue_type | 40 x breast_tissue, 34 x lymphocytes |
| GSE71118 | 117 | has_metastasis | 74 x No, 43 x Yes |
| GSE89292 | 46 | is_protected | 24 x FALSE, 22 x TRUE |
| GSE89565 | 57 | leukemia_type | 40 x AML, 17 x T-ALL |
| GSE99039 | 437 | disease | 233 x CONTROL, 204 x IPD |

**Supplementary Table 4.** *Test data MCC change from 90% train data to using 20% train data.*

| **dataset** | **gene-level** | **single PCA latent** | **single ICA latent** | **GPL570 ICA latent** | **AE latent** | **AVAE latent** | **mean** |
| --- | --- | --- | --- | --- | --- | --- | --- |
| GSE117239 | -0.025 | -0.129 | -0.131 | -0.039 | -0.118 | -0.041 | -0.081 |
| GSE131184 | -0.101 | -0.301 | -0.121 | -0.091 | -0.121 | -0.162 | -0.150 |
| GSE13159 | -0.036 | -0.044 | -0.059 | -0.047 | -0.041 | -0.070 | -0.049 |
| GSE14067 | -0.318 | -0.166 | -0.312 | -0.319 | -0.301 | -0.246 | -0.277 |
| GSE20685 | -0.146 | -0.148 | -0.118 | -0.198 | -0.134 | -0.169 | -0.152 |
| GSE20910 | -0.520 | -0.149 | -0.251 | -0.310 | -0.287 | -0.015 | -0.255 |
| GSE22459 | -0.035 | 0.028 | -0.216 | -0.064 | -0.147 | -0.011 | -0.074 |
| GSE28571 | -0.119 | -0.007 | -0.021 | -0.097 | 0.043 | -0.012 | -0.036 |
| GSE30674 | -0.180 | 0.010 | -0.124 | -0.149 | -0.160 | 0.045 | -0.093 |
| GSE30999 | -0.057 | -0.113 | -0.078 | -0.065 | -0.117 | -0.087 | -0.086 |
| GSE31189 | -0.235 | -0.025 | -0.170 | -0.189 | -0.256 | -0.216 | -0.180 |
| GSE33113 | -0.205 | -0.058 | -0.097 | -0.032 | -0.215 | -0.137 | -0.124 |
| GSE33630 | -0.049 | -0.242 | -0.063 | -0.048 | -0.026 | -0.058 | -0.081 |
| GSE35570 | -0.016 | -0.092 | -0.024 | -0.014 | -0.005 | -0.020 | -0.029 |
| GSE36059 | -0.212 | -0.148 | -0.111 | -0.211 | -0.262 | -0.121 | -0.178 |
| GSE37199 | -0.358 | -0.129 | -0.217 | -0.306 | -0.241 | -0.172 | -0.237 |
| GSE39582 | -0.092 | -0.086 | -0.163 | -0.106 | -0.084 | -0.121 | -0.109 |
| GSE48581 | -0.124 | -0.136 | -0.118 | -0.143 | -0.145 | -0.140 | -0.134 |
| GSE50006 | -0.060 | -0.141 | -0.180 | -0.062 | -0.026 | -0.101 | -0.095 |
| GSE51024 | -0.062 | -0.024 | -0.024 | -0.066 | -0.032 | -0.066 | -0.046 |
| GSE53757 | -0.020 | -0.023 | -0.014 | -0.020 | -0.022 | -0.029 | -0.021 |
| GSE56311 | -0.106 | 0.034 | 0.000 | 0.069 | -0.131 | -0.139 | -0.046 |
| GSE58812 | -0.074 | 0.043 | -0.036 | -0.147 | -0.177 | -0.136 | -0.088 |
| GSE60542 | -0.535 | -0.164 | -0.167 | -0.363 | -0.191 | -0.127 | -0.258 |
| GSE64951 | -0.092 | -0.155 | -0.233 | -0.230 | -0.189 | -0.057 | -0.159 |
| GSE66161 | 0.000 | 0.021 | -0.026 | 0.000 | 0.000 | 0.000 | -0.001 |
| GSE71118 | -0.111 | -0.151 | -0.147 | -0.196 | -0.137 | -0.162 | -0.151 |
| GSE89292 | -0.020 | -0.000 | -0.152 | -0.122 | -0.060 | -0.212 | -0.094 |
| GSE89565 | -0.219 | -0.288 | -0.186 | -0.227 | -0.252 | -0.219 | -0.232 |
| GSE99039 | -0.145 | -0.027 | -0.127 | -0.085 | -0.116 | -0.103 | -0.100 |
| mean | -0.142 | -0.094 | -0.123 | -0.129 | -0.132 | -0.103 |  |

**Supplementary Table 5.** *MCC performance comparison of regularization techniques on 90% train data using a paired samples Wilcoxon test.*

| **method** | **group1** | **group2** | **median difference** | **direction** | **p_value** |  | **p_value_adj** |
| --- | --- | --- | --- | --- | --- | --- | --- |
| gene-level | Lasso | Ridge | -0.00150 | group2 better | 0.35248 |  | 1.00000 |
| gene-level | Lasso | ElasticNet | -0.00668 | group2 better | 0.00975 |  | 0.02925 * |
| gene-level | ElasticNet | Ridge | -0.00034 | group2 better | 0.70712 |  | 1.00000 |
| single PCA latent | Lasso | Ridge | 0.25533 | group1 better | 4.4e-06 |  | 0.00001 *** |
| single PCA latent | Lasso | ElasticNet | 0.00079 | group2 better | 1.00000 |  | 1.00000 |
| single PCA latent | ElasticNet | Ridge | 0.26631 | group1 better | 1.4e-06 |  | 4.2e-06 *** |
| single ICA latent | Lasso | Ridge | 0.00480 | group1 better | 0.50276 |  | 1.00000 |
| single ICA latent | Lasso | ElasticNet | -0.00018 | group2 better | 0.20979 |  | 0.62937 |
| single ICA latent | ElasticNet | Ridge | 0.00890 | group1 better | 0.20541 |  | 0.61623 |
| GPL570 ICA latent | Lasso | Ridge | -0.00193 | group2 better | 0.20979 |  | 0.62937 |
| GPL570 ICA latent | Lasso | ElasticNet | -0.00206 | group2 better | 0.09875 |  | 0.29626 |
| GPL570 ICA latent | ElasticNet | Ridge | 0.00004 | group1 better | 0.44916 |  | 1.00000 |
| AE latent | Lasso | Ridge | -0.03188 | group2 better | 0.01289 |  | 0.03868 * |
| AE latent | Lasso | ElasticNet | -0.00548 | group2 better | 0.05994 |  | 0.17983 |
| AE latent | ElasticNet | Ridge | -0.00744 | group2 better | 0.01733 |  | 0.05199 |
| AVAE latent | Lasso | Ridge | 0.00108 | group1 better | 0.28936 |  | 0.86807 |
| AVAE latent | Lasso | ElasticNet | 0.00013 | group1 better | 1.00000 |  | 1.00000 |
| AVAE latent | ElasticNet | Ridge | -0.00020 | group2 better | 0.30949 |  | 0.92847 |

**Supplementary Table 6.** *AUC of proportion of the different representations for each dataset*

| **dataset** | **gene-level** | **GPL570 ICA latent** | **AE latent** | **AVAE latent** | **single PCA latent** | **single ICA latent** |
| --- | --- | --- | --- | --- | --- | --- |
| GSE50006 | 0.56 | 0.72 | 0.70 | 0.62 | 0.74 | 0.80 |
| GSE66161 | 0.71 | 0.80 | 0.77 | 0.84 | 1.00 | 0.95 |
| GSE20685 | 0.40 | 0.52 | 0.44 | 0.72 | 0.50 | 0.52 |
| GSE64951 | 0.15 | 0.12 | 0.27 | 0.28 | 0.59 | 0.97 |
| GSE36059 | 0.49 | 0.53 | 0.61 | 0.68 | 0.53 | 0.70 |
| GSE14067 | 0.50 | 0.52 | 0.47 | 0.62 | 0.47 | 0.81 |
| GSE33113 | 0.06 | 0.37 | 0.19 | 0.23 | 0.34 | 0.32 |
| GSE89565 | 0.50 | 0.62 | 0.64 | 0.60 | 0.67 | 0.67 |
| GSE117239 | 0.63 | 0.64 | 0.69 | 0.66 | 0.78 | 0.85 |
| GSE13159 | 0.55 | 0.69 | 0.72 | 0.70 | 0.61 | 0.86 |
| GSE89292 | 0.31 | 0.08 | 0.27 | 0.20 | 0.54 | 0.47 |
| GSE37199 | 0.45 | 0.47 | 0.38 | 0.60 | 0.50 | 0.81 |
| GSE31189 | 0.06 | 0.17 | 0.34 | 0.50 | 0.39 | 0.67 |
| GSE20910 | 0.44 | 0.11 | 0.37 | 0.41 | 0.52 | 0.55 |
| GSE30999 | 0.40 | 0.48 | 0.57 | 0.75 | 0.69 | 0.64 |
| GSE39582 | 0.55 | 0.61 | 0.65 | 0.63 | 0.58 | 0.85 |
| GSE22459 | 0.40 | 0.18 | 0.10 | 0.32 | 0.48 | 0.59 |
| GSE28571 | 0.17 | 0.17 | 0.43 | 0.28 | 0.52 | 0.34 |
| GSE131184 | 0.54 | 0.61 | 0.65 | 0.67 | 0.70 | 0.79 |
| GSE48581 | 0.42 | 0.52 | 0.59 | 0.61 | 0.49 | 0.74 |
| GSE71118 | 0.07 | 0.39 | 0.16 | 0.24 | 0.31 | 0.37 |
| GSE60542 | 0.49 | 0.55 | 0.27 | 0.40 | 0.53 | 0.63 |
| GSE53757 | 0.41 | 0.51 | 0.63 | 0.60 | 0.45 | 0.76 |
| GSE33630 | 0.50 | 0.56 | 0.54 | 0.69 | 0.68 | 0.79 |
| GSE56311 | 0.17 | 0.61 | 0.12 | 0.50 | 0.54 | 0.37 |
| GSE58812 | 0.10 | 0.09 | 0.44 | 0.39 | 0.32 | 0.26 |
| GSE51024 | 0.58 | 0.64 | 0.60 | 0.71 | 0.70 | 0.69 |
| GSE30674 | 0.14 | 0.06 | 0.14 | 0.28 | 0.44 | 0.62 |
| GSE99039 | 0.47 | 0.16 | 0.46 | 0.47 | 0.17 | 0.83 |
| GSE35570 | 0.66 | 0.69 | 0.74 | 0.71 | 0.87 | 0.86 |
| **median** | 0.45 | 0.52 | 0.47 | 0.60 | 0.53 | 0.69 |
| **mean** | 0.40 | 0.44 | 0.47 | 0.53 | 0.55 | 0.67 |

**SUPPLEMENTARY FIGURES**

**
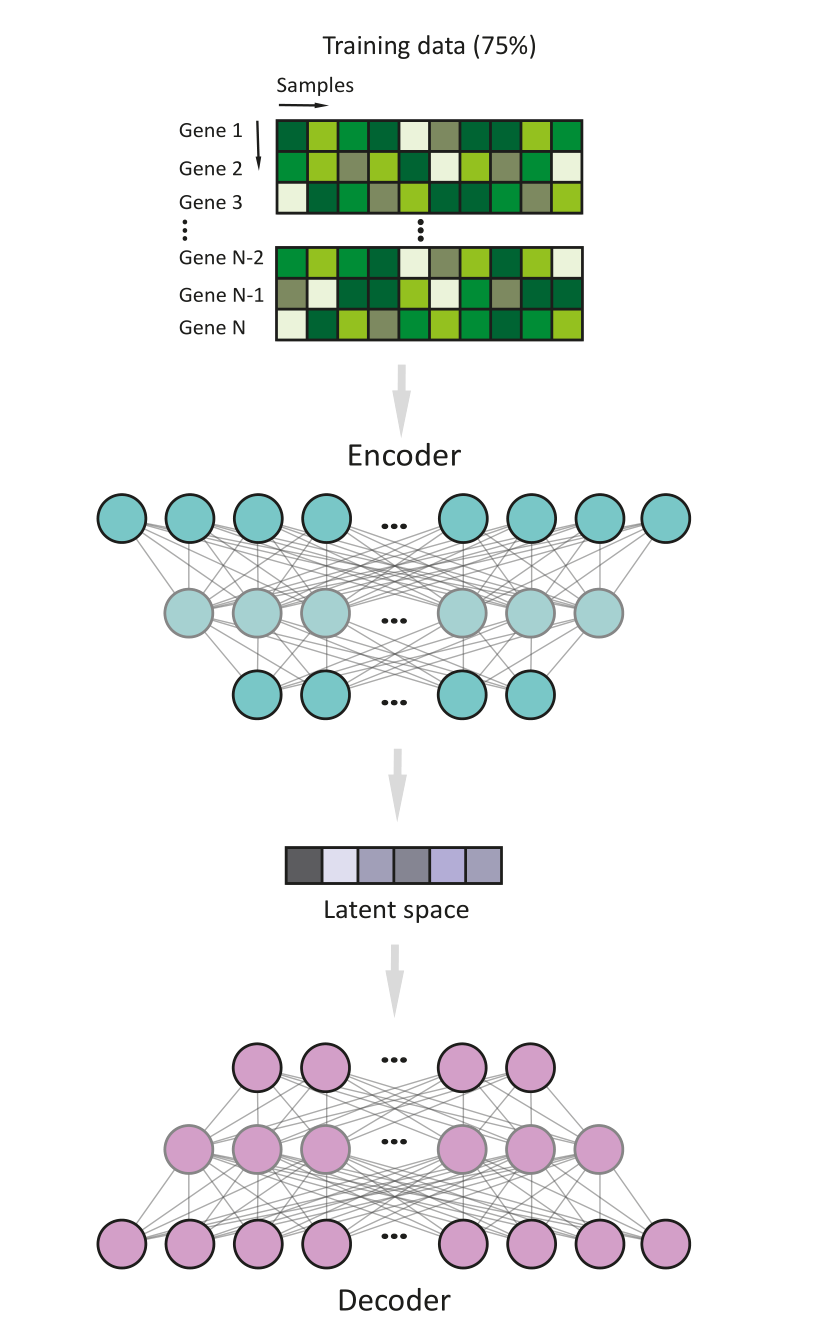
**

**Supplementary Figure 1.** *Schematic structure of AE network.* The AE consists of an encoder and a decoder. The detailed structure is described in Supplementary Table 2.


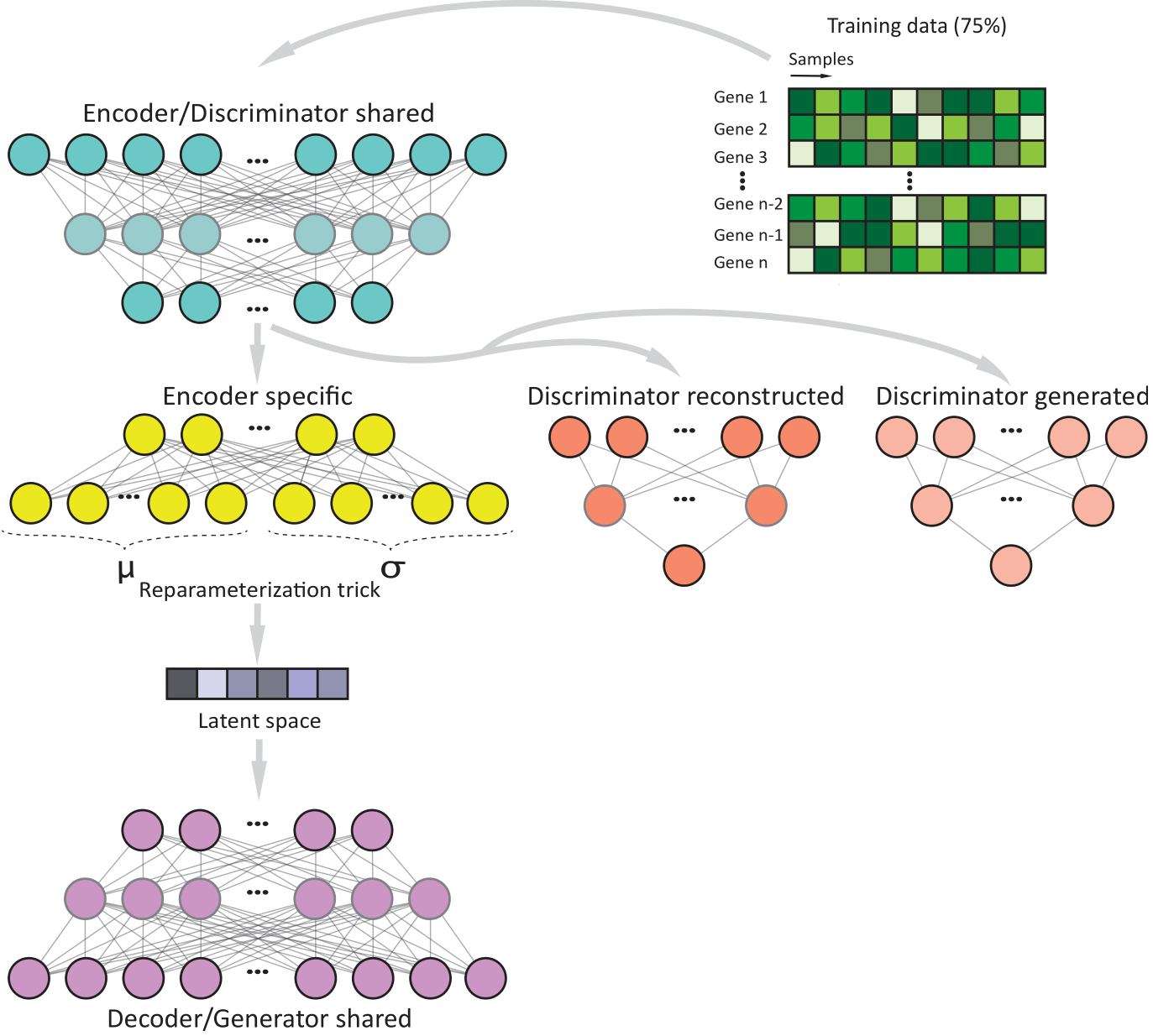


**Supplementary Figure 2.** *Schematic structure of AVAE method.* The AVAE consists of a shared encoder, specific encoder, decoder, and discriminators. The detailed structure is described in Supplementary Table 3.

*
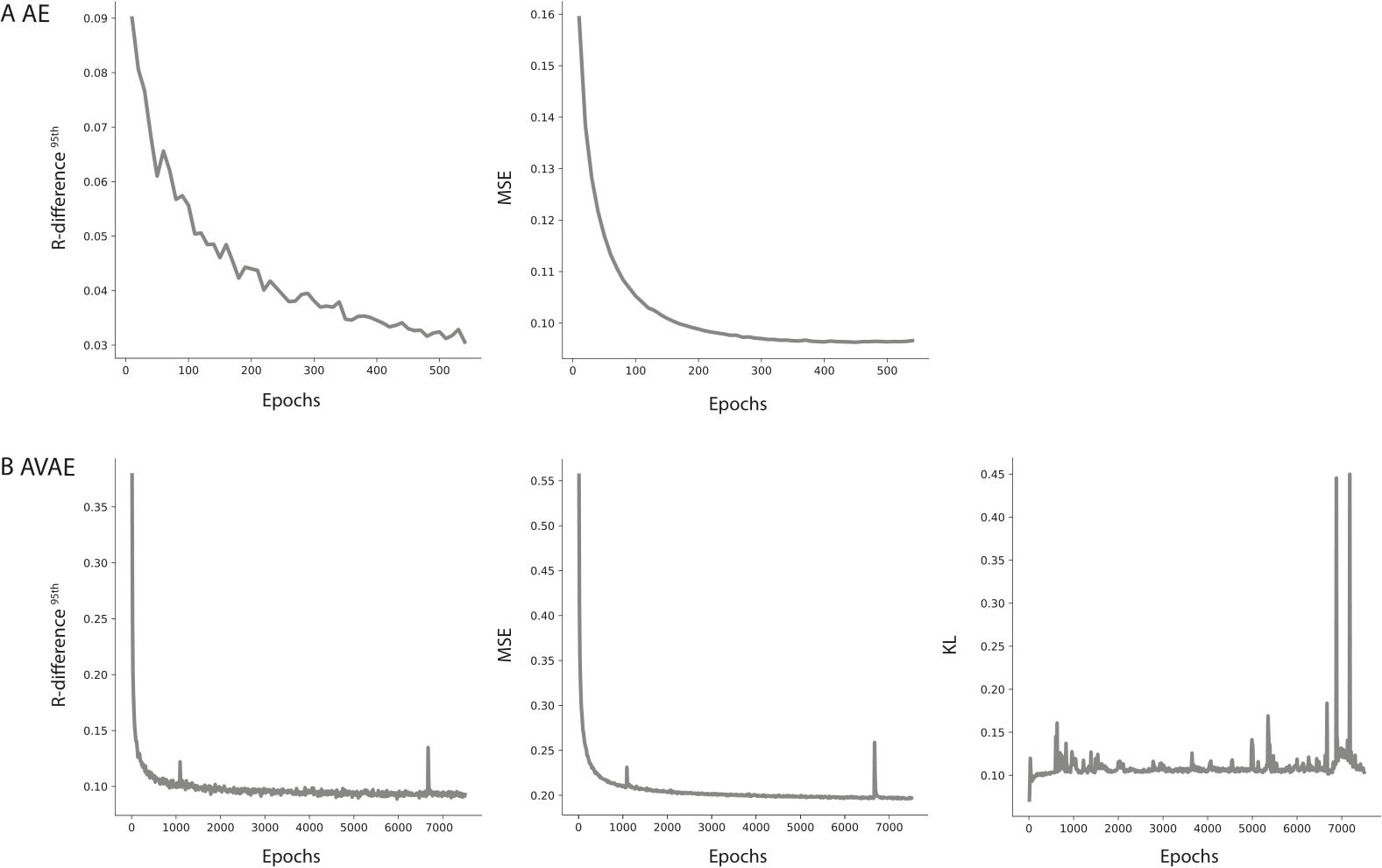
*

**Supplementary Figure 3.** *The learning curves of AVAE and AE networks on the validation set.* **A** The learning curves for mean squared error (MSE), R-difference^95th^ metrics on the AE network over 540 epochs. **B** The learning curves for MSE, R-difference^95th^, Kullback-Leibler (KL) metrics on the AVAE network over 7500 epochs. The R-difference^95th^ and the MSE show the reconstruction performance from the gene and sample perspective, respectively.


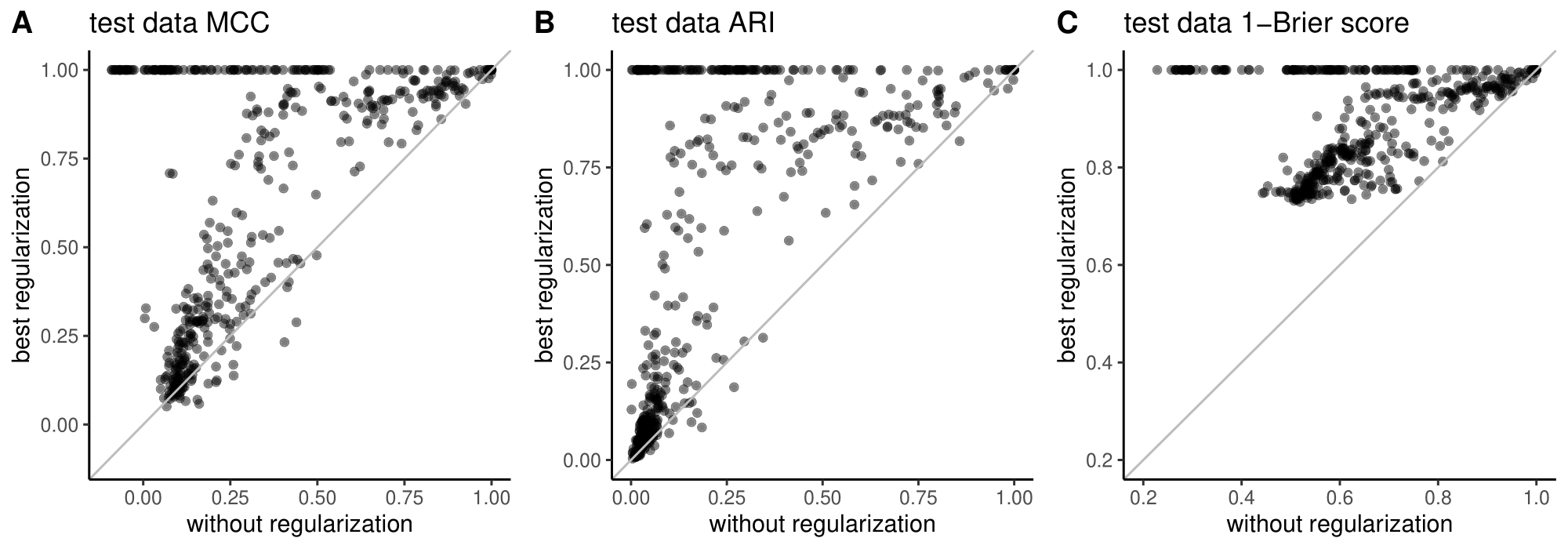


**Supplementary Figure 4.** *Comparative analysis of predictive model performances with and without regularization.* This figure illustrates the performance of predictive models applied to gene-level data and their latent representations across 30 datasets. The predictive performances of the two CV approaches (90% train with 10% test and 20% train with 80% test) were combined. The performance is displayed as different test data performances (**A**: Matthew correlation coefficient (MCC), **B**: Adjusted Rand Index (ARI), **C**: 1-Brier score) obtained through the CV-permutation test for both the predictive model with the best regularization technique and the model without regularization.


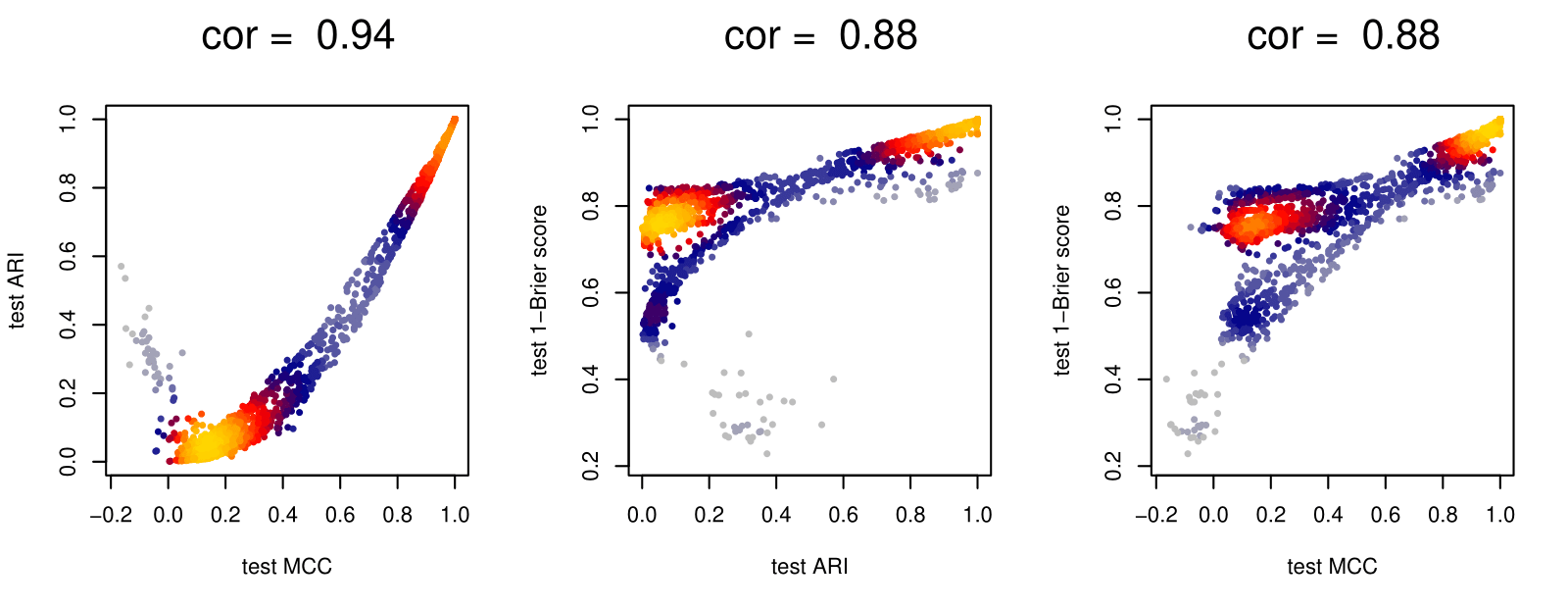


**Supplementary Figure 5.** *Predictive performance metrics comparison.* This figure shows how the Matthew Correlation Coefficient (MCC), the Adjusted Rand Index (ARI), and the 1-Brier Score behave relative to each other. The predictive performances for all 30 datasets of two CV approaches (90% train with 10% test and 20% train with 80% test) were combined. The Spearman correlation coeffiecient between two metrics is given.


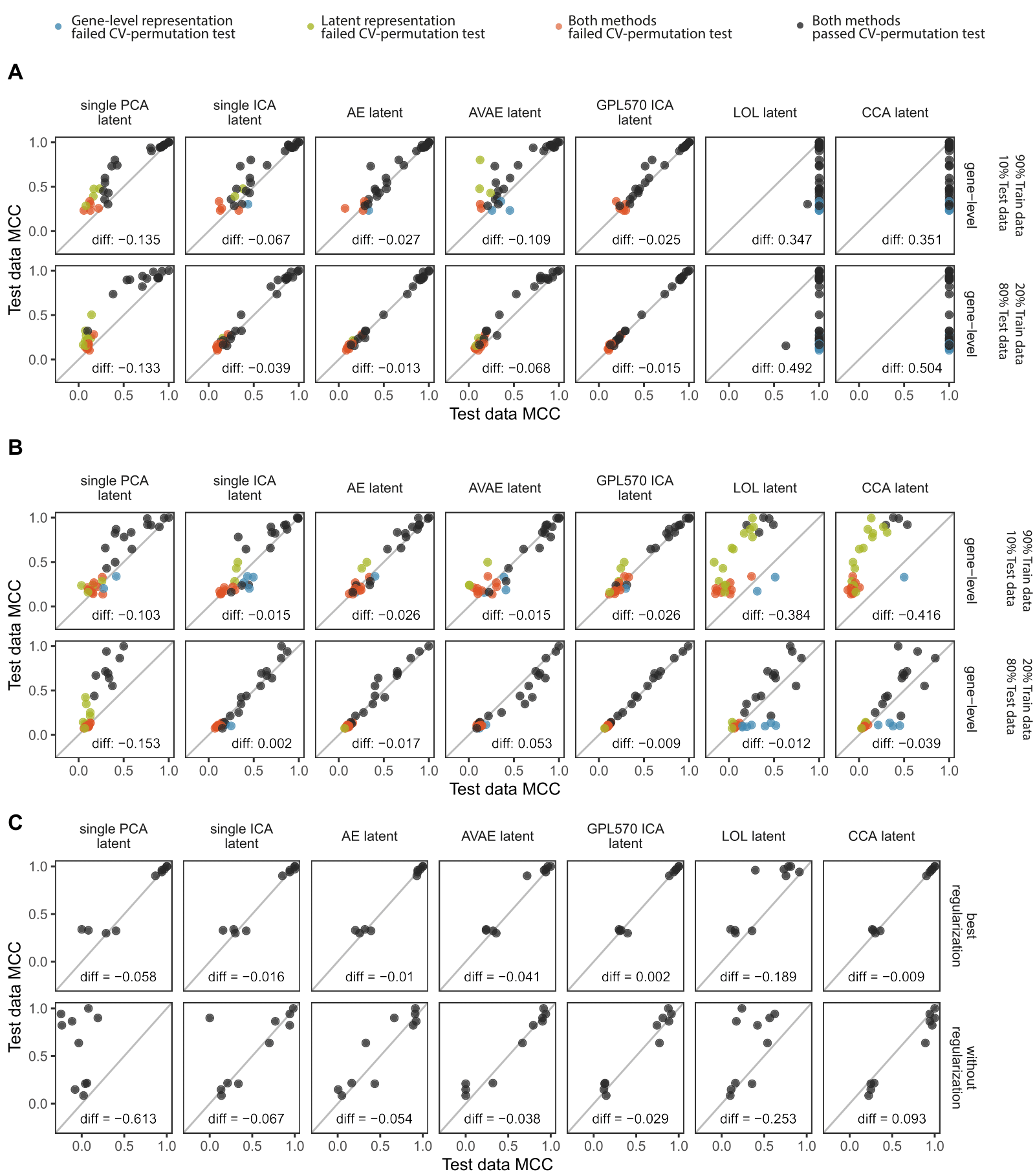


**Supplementary Figure 6.** *Comparative predictive performance of latent representations and gene-level representation.* Similar to Figure 3 A), the test Matthew correlation coefficient (MCC) is presented for all 30 datasets, showcasing the predictive performance of **A)** the optimal regularization technique and **B)** no regularization, as determined by the cross-validation-permutation test. The top row of each panel depicts predictive performance with 90% of the dataset for training, while the bottom row illustrates the performance with only 20% of the dataset allocated for training. The mean performance difference (diff) between gene-level and latent representations is provided, with a negative value indicating superior performance by the gene-level representation. **C)** The predictive performance of models with and without regularization is shown on five pairs of independent datasets for all latent representations in comparison to the gene-level representations. For each pair of datasets, one dataset was employed to train predictive models using regularization techniques, while the other dataset was used as test data for performance evaluation.


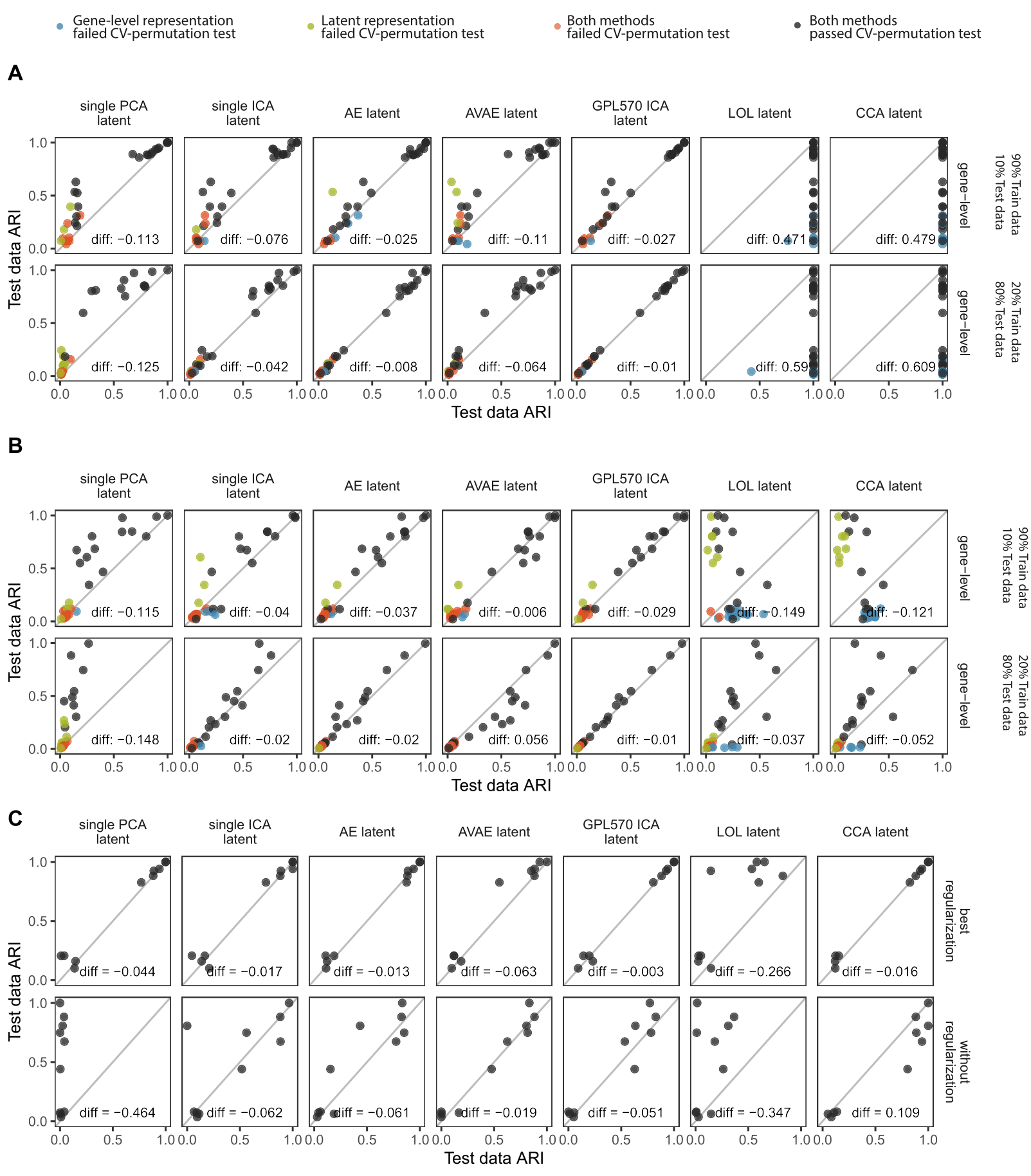


**Supplementary Figure 7.** *Comparative predictive performance of latent representations and gene-level representation.* Similar to Figure 3 A), the test Adjusted Rand Index (ARI) is presented for all 30 datasets, showcasing the predictive performance of **A)** the optimal regularization technique and **B)** no regularization, as determined by the cross-validation-permutation test. The top row of each panel depicts predictive performance with 90% of the dataset for training, while the bottom row illustrates the performance with only 20% of the dataset allocated for training. The mean performance difference (diff) between gene-level and latent representations is provided, with a negative value indicating superior performance by the gene-level representation. **C)** The predictive performance of models with and without regularization is shown on five pairs of independent datasets for all latent representations in comparison to the gene-level representations. For each pair of datasets, one dataset was employed to train predictive models using regularization techniques, while the other dataset was used as test data for performance evaluation.


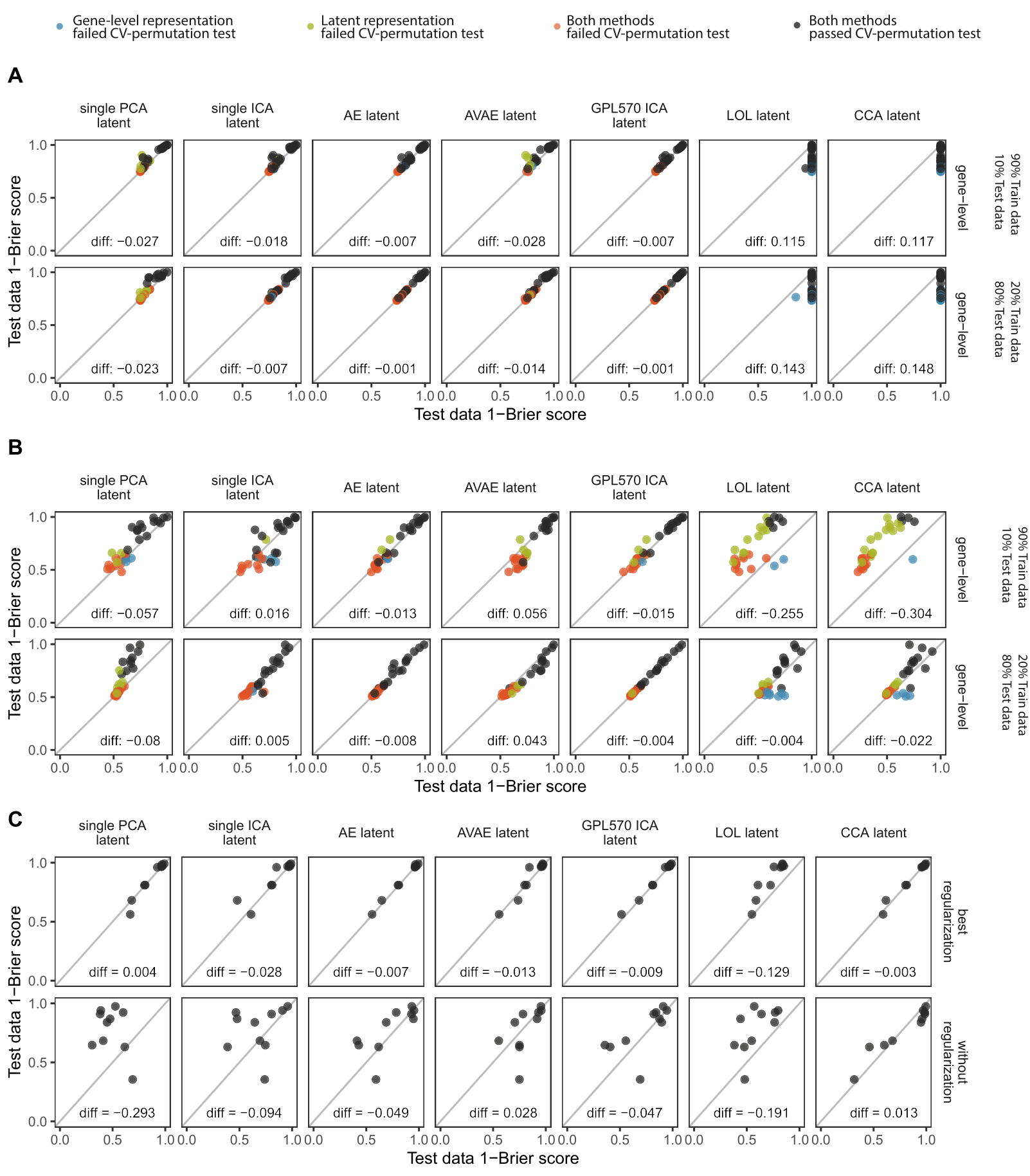


**Supplementary Figure 8.** *Comparative predictive performance of latent representations and gene-level representation.* Similar to Figure 3 A), the test 1-Brier Score is presented for all 30 datasets, showcasing the predictive performance of **A)** the optimal regularization technique and **B)** no regularization, as determined by the cross-validation-permutation test. The top row of each panel depicts predictive performance with 90% of the dataset for training, while the bottom row illustrates the performance with only 20% of the dataset allocated for training. The mean performance difference (diff) between gene-level and latent representations is provided, with a negative value indicating superior performance by the gene-level representation. **C)** The predictive performance of models with and without regularization is shown on five pairs of independent datasets for all latent representations in comparison to the gene-level representations. For each pair of datasets, one dataset was employed to train predictive models using regularization techniques, while the other dataset was used as test data for performance evaluation.


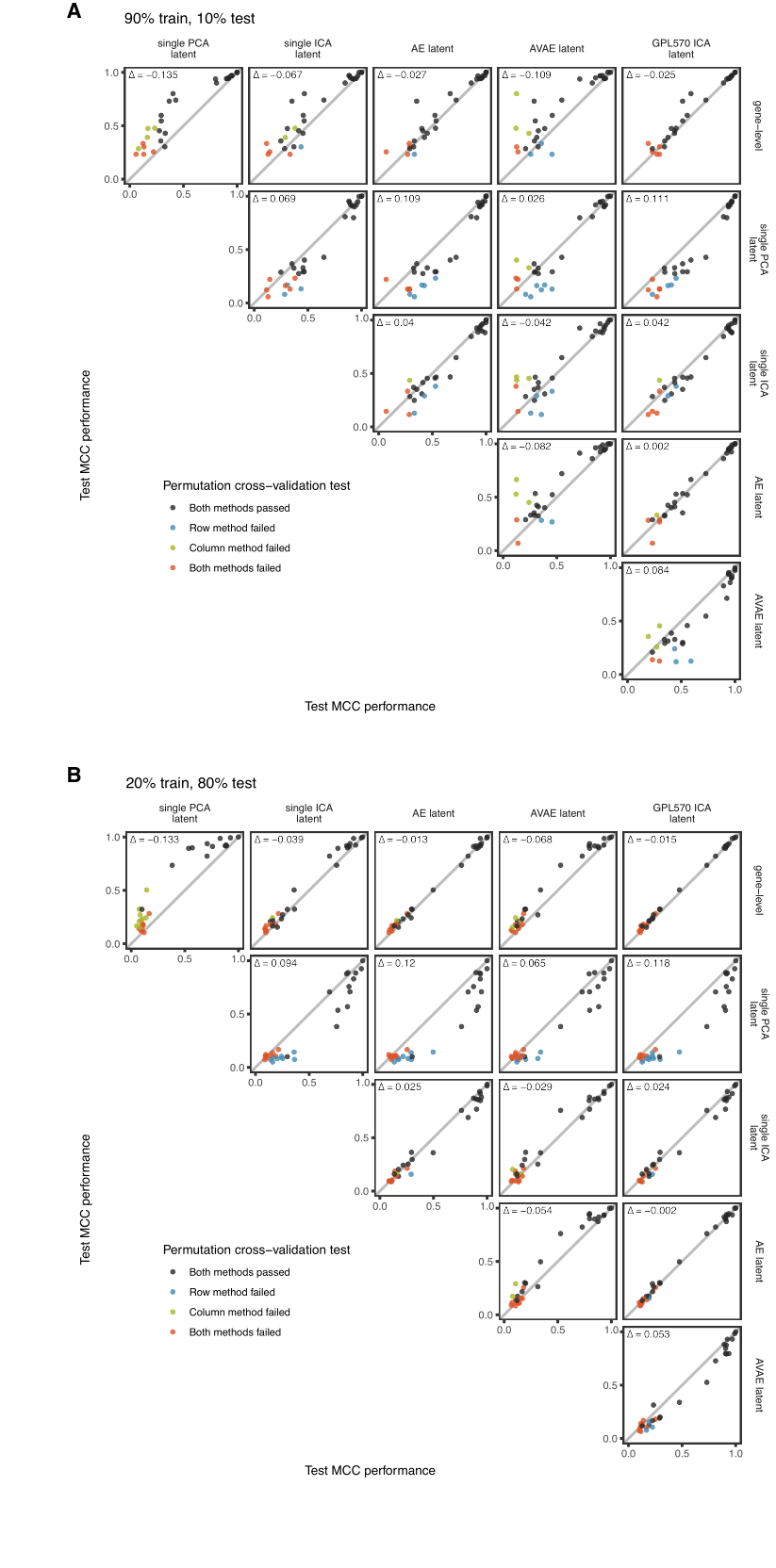


**Supplementary Figure 9.** *Pairwise comparison of the predictive performance between all different representations.* The test data MCC of the best regularization technique is shown for all 30 datasets. **A** Performance with 90% train and 10% test data. **B** Performance with 20% train and 80% test data.

**
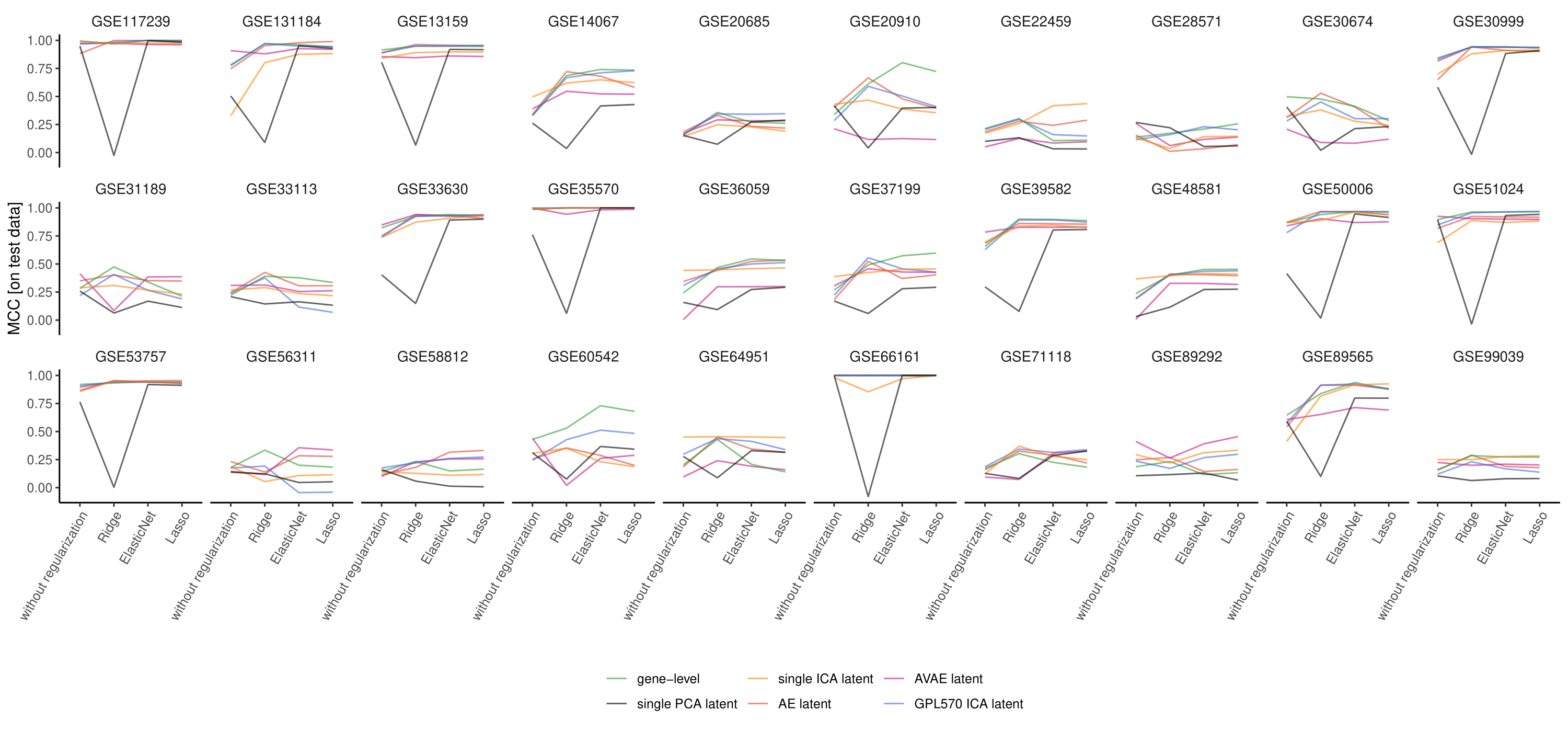
**

**Supplementary Figure 10.** *Predictive model performance across regularization technique and dataset.* The figure displays the performance of predictive models for each dataset, comparing the model without regularization to those employing Lasso, Ridge, and Elastic Net regularization techniques.


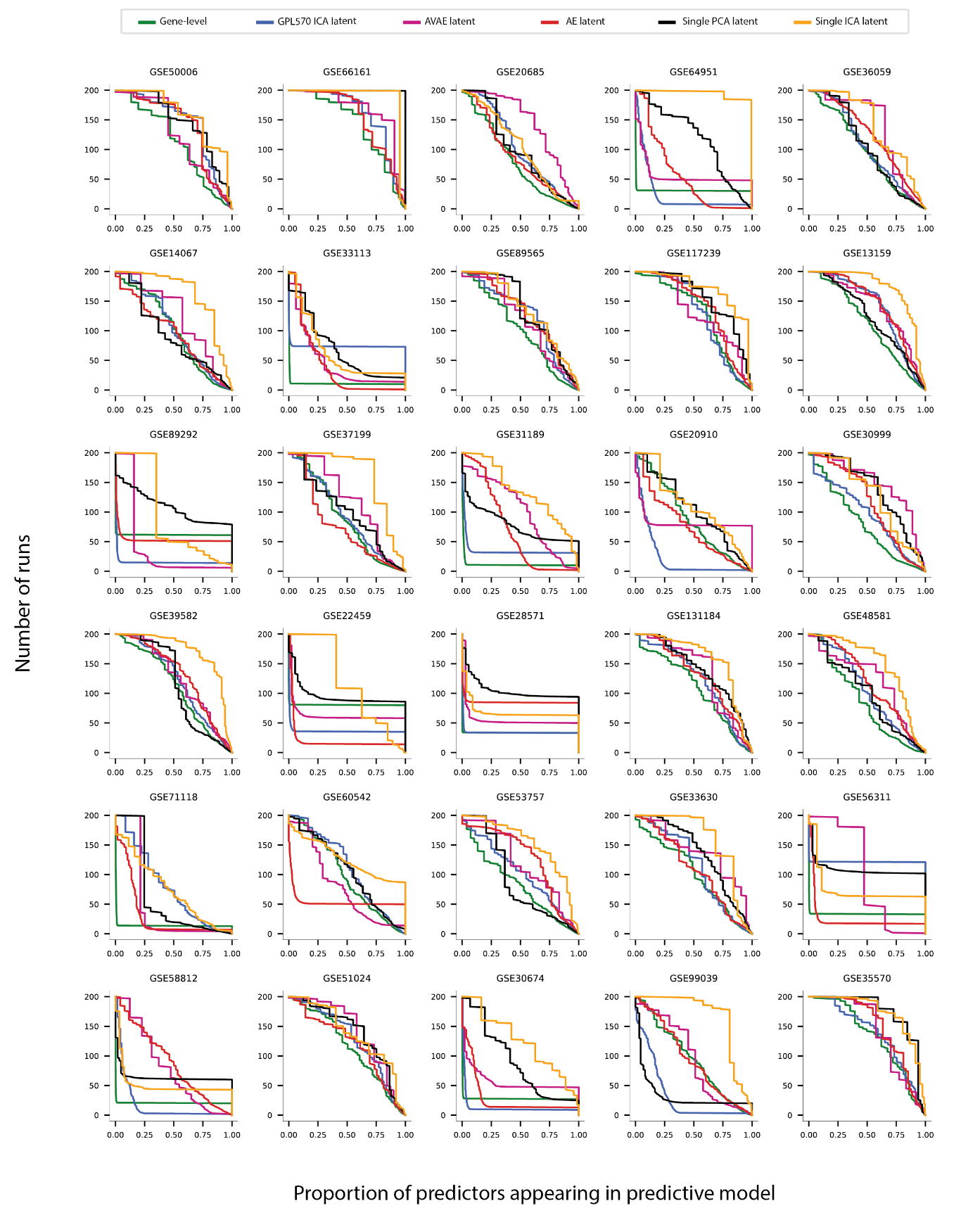
**Supplementary Figure 11.** *Robustness in predictor selection across representations per dataset.* This figure illustrates the robustness of predictor selection over 200 cross-validation runs. For each dataset, it shows in how many runs which proportion of the predictors were selected by Lasso. These curves define the predictor robustness as quantified by the Area Under the Curve (AUC) of the proportion of predictors, with a higher AUC indicating higher consistency and reliability in predictor selection.

**SUPPLEMENTARY NOTE**

**Data acquisition**

*An extensive compendium of transcriptomic profiles (GPL570 dataset)*

Publicly available microarray expression data generated with Affymetrix HG-U133 Plus 2.0 was obtained from the Gene Expression Omnibus (GEO; accession number GPL570) [1]. We downloaded all available raw files (CEL format). Because individual samples could be uploaded multiple times to GEO, we checked the samples for duplicates. We generated a message-digest algorithm 5 (MD5) hash for each raw data file that acts as a unique fingerprint. For each set of raw CEL files that share an identical MD5 hash, only one CEL file was retained for further analysis.

Preprocessing and aggregation of the CEL files were performed according to the robust multi-array average algorithm with apt-probeset-summarize of the Analysis Power Tools (release 2.11.3). We used custom CDF files (provided by BRAINARRAY, version 24) describing the layout for the processed Affymetrix gene expression arrays [2]. Within these custom CDF files, oligonucleotide probes on the Affymetrix platforms are reorganized based on updated genome and transcriptome information. Preprocessing was done with the standard method 'rma-sketch-gc-scale'. This standard method includes RMA style background adjustment, sketch quantile normalization, correcting feature intensity for variations in GC content, scaling CEL intensities, using unmodified perfect match probe intensities (no adjustment with mismatch probes), and performing a median polish to estimate target and probe effects.

PCA was applied to the sample Pearson product-moment correlation matrix of preprocessed expression profiles for quality control. The first principal component (PCqc) of such a correlation matrix between microarray generated samples expression profiles describes nearly always a constant pattern that dominates the data, explaining around 80-90% of the total variance. This pattern can be considered probe-specific or platform-specific variance, independent of the biological sample hybridized to the array. The correlation of each sample expression profile with this PCqc can be used to detect outliers, as arrays of lesser quality will have a lower correlation with the PCqc. We removed samples with a correlation of < 0.75.

Multiple probesets can target a single gene on the Affymetrix HG-U133 Plus 2.0 platform. We applied the R package 'jetset' (version 3.4.0) to obtain one-to-one mapping between genes and the 'best' probeset [3]. This package computes three scores for each probeset and then gets an overall score to determine the best probeset for the corresponding gene. **A)** Specificity score: - the specificity score is defined as the fraction of probes in a probe set that are likely to detect the targeted gene and unlikely to detect other genes. **B)** Coverage score: - the fraction of splice isoforms belonging to the targeted gene detected by the probeset is defined as the coverage score of the probeset. **C)** Robustness score: - the robustness score quantifies robustness against transcript degradation. The overall score for a probeset is the product of the specificity score, coverage score, and robustness score. The probeset with the highest overall score is considered the best probeset for a single gene and used in subsequent analyses.

This extensive compendium of transcriptomic profiles is referred to as the GPL570 dataset.

*Datasets used for training predictive models*

We selected datasets from GEO for training predictive models. All datasets were preprocessed as part of the GPL570 dataset described above. The sample annotation files with the phenotype information for the datasets were obtained using the R-package GEOquery v2.58.0.

**Training of the autoencoder (AE)**

Autoencoder (AE) methods are a type of unsupervised artificial neural network that can be applied to various tasks, such as feature learning, dimensionality reduction, data denoising, or classification. A basic autoencoder consists of an encoder and a decoder network. The encoder network will learn to compress the input data into representations with a limited number of 'new' variable (i.e., latent representation). The decoder network will learn to reconstruct the samples in the input data from their latent representations with minimal loss of information.

Here, the encoder maps the GPL570 dataset (139,786 samples x 19,863 genes) to the latent space (1,024 latent variables). The genes serve as the input variable for the encoder. The decoder tries to reconstruct the samples from their latent representation. A schematic representation of the AE is shown in Figure 1. A detailed description of hyperparameters and layers is shown in Supplementary Table 2.

After shuffling the samples in the input dataset, we split the dataset into a training (70%, n = 97,850), validation (15%, n = 20,968), and test set (15%, n = 20,968). Training of AEs is based on minimizing the difference between the input and reconstructed data. We used the mean squared error (MSE) loss function to train the encoder and the decoder parameters.

AE training was performed using Ranger [4], which combined Rectified Adam (RAdam) and Lookahead into a single optimizer [5,6]. RAdam stabilizes training at the start by using a warm-up technique on the learning rate to avoid early overfitting. The Lookahead mechanism improves learning robustness as training progresses and leads to faster loss convergence.

As described above, we used the MSE to train and evaluate the model's performance. MSE looks at the performance from a sample perspective. In addition, we obtained a metric that informed us of the reconstruction performance from the perspective of the genes. First, we calculated the Pearson correlation between the genes in the input data, resulting in a triangular correlation matrix with dimension n genes by n genes. Secondly, the same triangular correlation matrix was calculated with the reconstructed data. The absolute difference was calculated between the correlations obtained with the input and the reconstructed data for each gene-gene pair. After sorting the absolute correlation differences in ascending order, we obtained the 95th percentile (referred to as the R-difference^95th^ metric) as a reconstruction performance metric from the gene perspective. The lower the R-difference^95th^ metric, the better the reconstructed data captured the gene-by-gene correlation structure present in the input data.

During the training of the AE network, the performance for the validation set was calculated after every 10 epochs. In the lowest range of MSE and R-difference^95th^ metrics, when MSE did not increase anymore model was chosen as the final model (on epoch 540, Supplementary Data 1).

**Training of the adversarial variational autoencoder (AVAE)**

Adversarial variational autoencoder (AVEA) [7] combines the concepts of a variational autoencoder (VAE) [8] and a generative adversarial network (GAN) [9]. In GAN models, the concept is to generate new data from the noise that is indistinguishable from actual data. GANs consist of two sub-models, the generator, and the discriminator, which compete during training. While the generator tries to generate new samples that could have been drawn from the input dataset, the discriminator tries to predict if a sample came from the actual input dataset or came from the generator.

The concept of VAE is like the AE described above. The encoder of a VAE outputs parameters of a pre-defined distribution in the latent space for every input instead of outputting the actual variables in the latent space. Here, the VAE imposes a constraint on the latent distribution forcing it to be a normal distribution. The combination of VAE and GAN is called AVAE. AVAE has the encoder of VAE and discriminator of GAN. It is advantageous in performance on the generation and reconstruction over other state-of-the-art hybrid approaches [7].

AVAE learns a smooth latent state representation. This can lead to more meaningful biological representations and would allow, for example, better reconstruction of new transcriptomic profiles with similar properties by adding noise to the latent representation. A schematic representation of the AVAE is shown in Supplementary Figure 2. A detailed description of hyperparameters and layers is shown in Supplementary Table 3.

To train the AVAE, the GPL570 dataset was split into train (70%, n = 97,850), validation (15%, n = 20,968), and test set (15%, n = 20,968). During the training process, the AVEA tries to encode the input data and regenerate the closest possible representation. Additionally, the constraints of the AVAE force the latent distribution to adapt to the prior distribution. This tradeoff between the decoder reconstruction and the discriminator classification makes the training process challenging. For this, the optimizer Ranger with the same attributes as AE was used to simultaneously minimize two loss functions: MSE for the reconstruction performance and Kullback-Leibler divergence (KL) [10] to control the distribution of latent space.

The loss calculation for encoder and decoder is as follows: The encoder loss is equal to MSE loss of reconstructed data plus loss of the generated latent representation, calculated by KL. The decoder loss equals the discriminator loss of reconstructed plus the discriminator loss of generated data plus the MSE loss of reconstructed data. During AVAE network training, the performance for the validation set was calculated after every 100 epochs. In the lowest range of the MSE and R-difference^95th^ metrics, when MSE did not decrease anymore, the model was selected as the final model (on epoch 7500, Supplementary Data 2).

AE and AVAE were trained using Python with Tensorflow2 on GPU Quadro RTX 8000.

**Consensus independent component analysis on the GPL570 dataset**

Consensus independent component analysis (c-ICA) was performed to segregate the bulk transcriptomic profiles into statistically independent transcriptional components (TCs) as previously described in more detail [11].

In short, applying c-ICA on a gene expression dataset with p genes and n samples results in the extraction of i transcriptional components of dimension 1×p. Each TC captures the transcriptional footprint of an underlying process (e.g., biological process or cellular state). Each TC is composed of p scalars, representing the direction and magnitude of the effect of the underlying process on a gene's expression level. In addition to the TCs, c-ICA also provides a mixing matrix (MM) of dimension i×n, which contains coefficients representing the latent variable of a TCs in a sample. The inner product between the vector of 'coefficients' of an individual sample in the MM and the vector of scalars per individual gene over all TCs results in reconstructed mRNA expression profiles as close to the input profiles.

First, a preprocessing technique called whitening is applied to the input dataset to make the convergence rate of the ICA algorithm faster. Whitening transforms the input dataset into a new dataset (where all n transformed samples are orthogonal). Next, ICA was conducted on the whitened mRNA expression dataset, resulting in the extraction of 𝑖 independent components. To choose 𝑖, PCA was conducted on the sample-by-sample covariance matrix. The number of principal components capturing at least 90% of the total variance in GPL570 dataset was used as input components for the c-ICA. c-ICA was performed on the whitened dataset using an in-house C++ implementation of the FastICA algorithm. FastICA was performed with the following parameters: contrast function = hyperbolic tangent, stopping criteria (epsilon) = 0.0001, proportion of sample to use in an iteration = 1.0, stabilization mode = enable, finetune mode = enable, step size (mu) in stabilization mode = 0.75.

Due to the optimization algorithm that is randomly initialized, the FastICA algorithm can get stuck in a local minimum of the search space. The resulting TCs can be different for different random initializations of the FastICA algorithm. A consensus sources estimation (CSE) algorithm can be used to get a set of TCs for which the probability of converging to a local minimum is minimized. CSE assumes that over many runs of ICA, the algorithm does not converge to any local minima for most of the runs. In the present study, CSE was conducted on 25 different runs of ICA, each with a different random initialization. The CSE algorithm consists of the following steps:

First, the TCs from all runs are combined into a single matrix. In this matrix, the Pearson correlation between all TCs is calculated, resulting in a triangular correlation matrix with dimension m by m TCs. Then, the TCs are clustered based on having a correlation > 0.9. Each cluster represents a group of TCs which are recoverable throughout multiple runs. Per cluster, the medoid TC (based on distance correlation with all other cluster members) was selected as the winner. The winning TCs, which were present in at least 20% of the 25 different ICA runs, were selected for the final set of consensus TCs.

These TCs yield an output matrix of p genes by m components, each covering a robust, statistically independent, and distinct footprint of an underlying process.

**Dimensionality reduction methods applied to single datasets used for predictive modeling**

Predictive models that used the preprocessed gene expression data from each dataset as input were considered the reference for comparing predictive performance. They are included without dimensionality reduction and contain the highest feature complexity due to the high number of genes. This gene expression data is further referred to as gene-level representation. This gene-level representation served as input to several dimensionality reduction methods described below.

*Supervised dimensionality reduction*

Supervised dimensionality reduction methods can be used to obtain a lower dimensional representation of gene expression data while preserving the discriminating information, such as the respective phenotypes in our datasets. We applied Linear Optimal Low-Rank Projection (LOL) [18] and Low-Rank Canonical Correlation Analysis (CCA) [19] on the gene-level datasets with their corresponding phenotypes. These methods were run using the functions "lol.project.lol()" and "lol.project.lrcca()" from the R package lolR v2.1. The resulting representations are referred to as supervised LOL- and CCA latent representations, respectively.

*Principal component analysis*

A commonly used method to reduce the dimensionality of gene expression data is principal component analysis (PCA). PCA is a method to condense a multi-dimensional dataset into a set of lower dimensions to reveal the simplified linear structure of the data that often underlies it. In this study, PCA represents a transformation of a set of correlated samples into a set of uncorrelated 'new' samples containing principal components (PCs). Each PC could be interpreted as a feature.

PCA was applied using the function "prcomp()" (R version 4.0.3) and taking the output x of this function, the matrix of the rotated data. In detail, for each gene-level representation, the genes were standardized to a mean of 0 and a standard deviation of 1 before the sample correlation matrix was calculated. The eigenvectors and eigenvalues of the sample correlation matrix were subsequently calculated. The eigenvalues describe the variance each PC captures from the input data. The inverse of the eigenvectors is the linear transformation of the original samples to the PCs. The coefficients of the inverse of the eigenvector matrix indicate the relative weight of each sample in the PCs. They can be interpreted as a "latent variables" measurement of the feature that the PC describes. The activity scores of the PCs capturing 100% of the variance observed in the dataset were used as input for predictive modeling and are referred to as the single PCA latent representation throughout this manuscript.

*Consensus independent component analysis*

The gene-level datasets were entered separately into the c-ICA implementation described previously. For each dataset, the principal components that could explain 100% variance in the whitening step were the input components for ICA. Next, the FastICA algorithm was performed with the following parameters: contrast function = hyperbolic tangent, stopping criteria (epsilon) = 0.00001, proportion of sample to use in an iteration = 1.0, stabilization mode = enable, finetune mode = enable, step size (mu) in stabilization mode = 0.5. CSE was determined for 50 different ICA runs. The resulting consensus mixing matrix, consisting of samples and their independent component variable, is used as input variable for further predictive modeling and is referred to as a single ICA latent representation.

*Autoencoder*

Each of the gene-level datasets was provided as input separately into the AE network trained on the GPL570 dataset. The datasets were passed through the encoder layers of the AE network to obtain a dimensionality-reduced representation. This representation contains samples that have 1,024 latent variables. We will refer to this representation of each dataset as their corresponding AE latent space.

*Adversarial variational autoencoder*

The gene-level datasets were provided as input separately into the trained AVAE network. Each input dataset was subsequently passed through the shared encoder and the specific encoder (see Supplementary Figure 2). This yielded in an output layer that contained 256 latent variables divided into a mean and a standard deviation part. Then, the mean-vector was taken from the previous output layer, which had 128 latent variables for each sample. We refer to this representation as AVAE latent representation.

*c-ICA transformation obtained with the GPL570 dataset*

The gene-level datasets (X_p x m_ with m samples and p = 19,863 genes)
were projected into a lower-dimensional representation using the c-ICA transformation obtained with the GPL570 dataset. This representation of dataset X is referred to as the projected mixing matrix (pMM_m x i_ with i = 3,286 TCs from the GPL570 c-ICA), which can be inferred from the independent component matrix (S_i x p_). Using the pseudo-inverse solution from [12], the pMM can be calculated as follows:


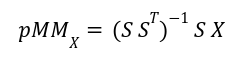


The pMM of a dataset containing the independent component variables in the samples is referred to as GPL570 ICA latent representation in this manuscript.

In summary, gene-level datasets were individually projected into their dimensionality-reduced versions using PCA and c-ICA trained on the corresponding dataset itself, as well as AE, AVAE, and c-ICA trained on the GPL570 dataset. The gene-level datasets and these latent representations were used as input for the predictive modeling part.

**Predictive modeling**

*Evaluation metrics for performance*

The maximum Matthew correlation coefficient [13] (MCC) was chosen for logistic regression as a comparative measure of model performance. This measurement proved advantageous over the area under the receiver operating characteristic curve (AUROC) in the case of imbalances in the phenotypic labels of datasets [13]. All the following predictive modeling analysis was performed using R v4.0.3. For calculating MCC, the measure "mat" from the "prediction" function's performance object was used (package ROCR v1.0-11). To consider the clustering of predicted phenotypes, we also included the Adjusted Rand Index [20] and the Brier score [21]. These values were obtained using the maximum value from the "adjustedRandIndex()" function for each split and the "BrierScore()" function from the R package mclust v6.0.0.

*Splitting in training and test set*

To evaluate the performance of the predictive models, each dataset was split into a train and test set. A random split may result in an imbalance of phenotypic labels in these sets, up to the point that one label group is not present in the train set. To avoid this, the division into a train and test set was done in a stratified manner on the phenotypic labels. The stratified split was done using the "create_folds" function with the stratified parameter from splitTools package v0.3.1.

*Cross-validation*

We used a k-fold cross-validation (CV) to validate the robustness of the predictive models. First, the samples of a predictive dataset were split into k-folds in a stratified manner as described previously. Then, k-1 folds were used as a training set and validated using the remaining k_th_ fold as the test set. After CV, the global model performance was calculated using the combined predictive labels of all folds. CV was performed in two ways, with k=10 (90% train, 10% test) and a reverse scheme of k=5 (20% train, 80% test) to observe the predictive behavior with a smaller training sample size.

*Permutation test*

A phenotype permutation test based on 200 permutations was performed to assess the significance of the predictive model performance (i.e., CV-permutation test). Per dataset, the phenotypic labels were randomly shuffled before performing CV. For each random reshuffling, the predictive model calculated the cross-validated evaluation metrics, providing a null distribution of the model performance. The tail area of this null distribution beyond the evaluation metric obtained from the actual data was the permutation significance level for testing the null hypothesis of no association between the dataset and the phenotypic label. The statistical significance is described by the p-value, defined as the number of permutations that are equal or exceed the mean of the actual performance, divided by the number of total permutations. The minimum reachable p-value is 1/number of total permutations.

*Regularization techniques*

For each of the representations, regression models with three regularization techniques were trained: Lasso, Ridge, and Elastic Net. Many correlated predictors, known as multicollinearity, can lead to unstable regression models and misestimating of the predictors' relevance to the response. By penalizing the predictors' coefficients, regularization techniques try to reduce the effects of multicollinearity. This penalizing can lead to a more accurate estimation of regression models. By adding a penalty factor to the cost function (*c*), the regularization techniques discourage learning a complex model with inter-correlated predictors but focus on a more stable model with less multicollinearity.

The following three regularization techniques were used:

*Lasso regularization*

Lasso [14] minimizes the following penalized version of *c* to estimate the coefficients β corresponding to all the predictors of the model:


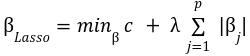


The coefficients β get penalized using the absolute coefficient weights, whereas λ defines the impact of penalization. Due to Lasso, coefficients of a subset of multicollinear predictors would be estimated as zero. Hence, Lasso yields a sparse model, allowing a more straightforward interpretation of the model through variable selection.

*Ridge regularization*

For Ridge [15], *c* is altered by adding a penalty equivalent to the square of the magnitude of the coefficients:


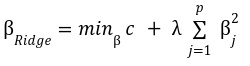


The coefficients β can approach zero but will never be equivalent to zero. Thus, Ridge will set coefficients of a subset of multicollinear predictors close to zero.

*Elastic Net regularization*

Elastic Net [16] combines Lasso and Ridge and tries to find the most optimum proportion of combination to take advantage of both methods:


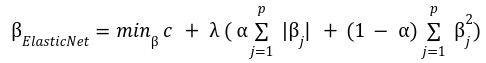


If α=1, Elastic Net is equivalent to Lasso. If α=0, it is identical to Ridge. Through refining α, it tries to find the best tradeoff between both methods.

The regularization techniques were conducted in R with the glmnet package v4.1-1 [17]. In the scripts, the default parameters were used with alpha=0, alpha=1, and alpha=0.5, for Ridge, Lasso, Elastic Net, respectively. The standardization of the input data is applied within glmnet.

The impact of the penalization is controlled by λ. If λ is too high, the model will have a lower degree of freedom with more coefficients close to zero, resulting in underfitting. An underfitted model will not learn enough about the training data to make accurate predictions. If λ is too low and approaches zero, the model will be more complex, and the risk of overfitting the data emerges. λ is determined separately by cross-validating several predictive models and finding the optimal lambda on these runs. For the CV step, stratified sampling was used to split the samples into 10-folds. Then, based on these folds, the "cv.glmnet" function was used to determine the best λ parameter (lambda.min). Then, the selected λ was used to fit the final predictive model with the function "glmnet". The final predictive model performance is the mean out of 200 10-fold CV runs.

*Independent datasets used to validate predictive models*

We used five pairs of independent datasets from GEO, each pair exhibiting the same phenotype. These phenotypes include kidney rejection (GSE48581 and GSE22459), psoriasis lesion vs. normal distinction (GSE30999 and GSE117239), leukemia type differences (GSE13159 and GSE131184), thyroid cancer vs. normal distinction (GSE35570 and GSE33630), and breast cancer metastasis event occurrence (GSE20685 and GSE58812). For single PCA and single ICA, the test dataset was standardized by the mean and standard deviation of the train data before projecting the test data to the dimensionality reduced representation using the PCs or TCs. For each dataset pair, one dataset is used to train predictive models with regularization techniques, while the other dataset serves as test data for performance evaluation.

**Robustness of predictors**

The predictor importance of the predictive models depends on the choice of λ. The a priori selection of λ is affected by the randomness of the CV sampling, resulting in a different set of predictors being selected for each prediction model.

The robust selection of predictors was checked using a cross-validation approach. Each dataset's samples were split 20 times in a stratified manner into k=10 folds. k-1 folds served as input data for predictive modeling with Lasso for each fold. For each predictor, the frequency of occurrence in 20 distinct 10-fold predictive models (overall 200 runs) was evaluated to investigate its robustness. The different representations of datasets have different numbers of predictors. Hence, the proportion of predictors that appear in the predictive model to the total number of predictors is used as a measure of robustness. The overall performance comparison of various methods is determined by calculating the area under the curve of the proportion of predictors to number of runs (AUC of proportion), for each individual dataset.

**REFERENCES**

1. Barrett T, Wilhite SE, Ledoux P, et al. NCBI GEO: archive for functional genomics data sets--update. Nucleic Acids Res 2013; 41:D991-995
2. Dai M, Wang P, Boyd AD, et al. Evolving gene/transcript definitions significantly alter the interpretation of GeneChip data. Nucleic Acids Res 2005; 33:e175
3. Li Q, Birkbak NJ, Gyorffy B, et al. Jetset: selecting the optimal microarray probe set to represent a gene. BMC Bioinformatics 2011; 12:474
4. Wright L. Ranger - a synergistic optimizer. GitHub repository 2019;
5. Liu L, Jiang H, He P, et al. On the Variance of the Adaptive Learning Rate and Beyond. 2021;
6. Zhang MR, Lucas J, Hinton G, et al. Lookahead Optimizer: k steps forward, 1 step back. arXiv:1907.08610 [cs, stat] 2019;
7. Munjal P, Paul A, Krishnan N. Implicit Discriminator in Variational Autoencoder. 2020 International Joint Conference on Neural Networks (IJCNN) 2020; 1–8
8. Kingma DP, Welling M. Auto-Encoding Variational Bayes. 2022;
9. Goodfellow IJ, Pouget-Abadie J, Mirza M, et al. Generative Adversarial Networks. 2014;
10. Hershey JR, Olsen PA. Approximating the Kullback Leibler Divergence Between Gaussian Mixture Models. 2007 IEEE International Conference on Acoustics, Speech and Signal Processing - ICASSP ’07 2007; IV-317-IV–320
11. Bhattacharya A, Bense RD, Urzúa-Traslaviña CG, et al. Transcriptional effects of copy number alterations in a large set of human cancers. Nat Commun 2020; 11:715
12. Chiappetta P, Roubaud MC, Torrésani B. Blind source separation and the analysis of microarray data. J Comput Biol 2004; 11:1090–1109
13. Chicco D, Jurman G. The advantages of the Matthews correlation coefficient (MCC) over F1 score and accuracy in binary classification evaluation. BMC Genomics 2020; 21:6
14. Tibshirani R. Regression Shrinkage and Selection via the Lasso. Journal of the Royal Statistical Society. Series B (Methodological) 1996; 58:267–288
15. Hoerl AE, Kennard RW. Ridge Regression: Biased Estimation for Nonorthogonal Problems. Technometrics 1970; 12:55–67
16. Zou H, Hastie T. Regularization and Variable Selection via the Elastic Net. Journal of the Royal Statistical Society. Series B (Statistical Methodology) 2005; 67:301–320
17. Friedman JH, Hastie T, Tibshirani R. Regularization Paths for Generalized Linear Models via Coordinate Descent. Journal of Statistical Software 2010; 33:1–22
18. Vogelstein JT, Bridgeford EW, et al. Supervised dimensionality reduction for big data. Nature Commun 2021; 12.1:2872
19. Shin H, Eubank RL. Unit canonical correlations and high-dimensional discriminant analysis. J Stat Comput Simul 2011; 81:167-178
20. Hubert L, Arabie P. Comparing Partitions. J Classif 1985; 2:193–218
21. Brier GW. Verification of forecasts expressed in terms of probability. Mon Weather Rev 1950; 78.1:1-3
